# Clinical implementation of patient-specific quality assurance for synthetic computed tomography

**DOI:** 10.1016/j.phro.2025.100764

**Published:** 2025-04-04

**Authors:** Francesca Nella, Stephanie Tanadini-Lang, Riccardo Dal Bello

**Affiliations:** Department of Radiation Oncology, University Hospital Zurich and University of Zurich, Zurich, Switzerland

**Keywords:** MR-only, Synthetic CT, Quality assurance, PSQA, Clinical implementation, Pelvis, Brain, Commissioning, Acceptance criteria

## Abstract

**Background and purpose:**

In a magnetic resonance (MR) only planning workflow, MR image is the sole dataset acquired. In order to calculate the dose deposition, a synthetic CT (sCT) is generated to substitute the planning computed tomography (CT). This study aimed to establish acceptance criteria for the clinical implementation of patient-specific quality assurance (PSQA) for sCT.

**Materials and methods:**

A retrospective study was conducted on 60. 30 patients underwent a CT scan in treatment position and an MR in diagnostic position. 30 patients had both CT and MR images acquired in treatment position. For the latter group, a sCT for dose calculation was generated and compared against three PSQA methods: recalculation on (A) water override of the body, (B) tissue classes with bulk density overrides and (C) planning CT. The relative dose differences (ΔD [%]) between the sCT and the PSQA methos were evaluated.

**Results:**

ΔD for PTV Dmean for method (A) were within 3% for pelvis and 4% for brain cohorts, with standard deviations below 1%. Methods (B) and (C) remained within 2% and 1%, respectively, with deviations up to 1%.

**Conclusion:**

The present study proposes a robust PSQA method for MR-only planning. Method (A) is a valuable tool for identifying potential large outliers for Dmean deviations (> 5 %) and it is proposed as the routine PSQA. Method (B) can be used for pelvis cases to improve detection to the 2 % level if method (A) fails. If both (A) and (B) fail, method (C) can be used as a fall-back.

## Highlights

• We implement synthetic CT in clinical use and report our experience.
• We develop an automated patient-specific quality assurance strategy.
• The proposed approach is applicable to detect large outliers.
• A more sophisticate method can refine the detection but requires manual input.
• The fall-back approach in case of failure requires a planning computed tomography (CT) scan.

## 1 Introduction

Computed tomography (CT), magnetic resonance (MR), and photon emission tomography (PET) have become indispensable modalities for treatment planning and delivery [1], [2]. While CT provides electron density (ED) information necessary for dose calculation, MR offers enhanced soft tissue contrast, making it particularly advantageous for delineating targets and organs at risk (OARs) [2], [3]. Consequently, MR is now established as imaging modality for tumor sites such as prostate [4], brain [5], gynecological region [6] and rectum [7]. Nevertheless, challenges persist with MR, including susceptibility to motion artefacts, geometric distortions [8], [9], limitations in reproducing cortical bone [10], and its lack of quantitative information [10].

The term “synthetic CT” (sCT) refers to CT images generated from MR data using various methods. In deep learning (DL), convolutional neural networks (CNNs), which learn direct MR-to-CT image mapping, have demonstrated superior accuracy and quality of the resulting sCT images compared to previously proposed techniques such as tissue segmentation-based or atlas-based approaches [3], [11], [12]. As network input, the MR sequence T1 VIBE Dixon, which exploits the chemical shift between water and fat, can be reconstructed into four different contrasts: in-phase, opposed-phase, water-only and fat-only images, making it beneficial for sCT generation [11].

MR-only planning, eliminates the need for a planning CT, offering key advantages: it eliminates systematic MR-CT registration uncertainties [13], [14], [15], reduces patient hospital visits and streamlines workflows [1], [3], [9], [16]. However, geometric distortions inherited by the sCT [3], and the patient’s contraindications to MR must be considered [17].

Dosimetric deviations based on various dose-volume histograms (DVH) parameters between CT and sCT generated via DL methods are generally within 1 % for the pelvis and brain [11], [18], meeting the 2 % clinical applicability threshold proposed by Korsholm et al, assuming a 1 % dose calculation error when using CT [19].

Although commercial solutions for sCT generation are available for various anatomical sites, quality assurance (QA) methods are still under development [17], [20], leading to institution-specific approaches which vary due to lack of consensus and guidelines [1]. Since end-to-end verification is not feasible, alternative methods for verifying dose distribution, with different degrees of complexity and accuracy, should be investigated.

In MR-only planning, independent dose calculations on various ED maps may complement conventional QA processes. This work aims to evaluate and clinically implement automated PSQA techniques for sCT, focusing on accuracy and robustness through a comprehensive retrospective analysis of MR-only planning for brain and pelvis.

## 2 Materials and methods

### 2.1 Treatment Preparation

The study was structured into two phases: preparatory and commissioning. The first aimed to establish an efficient workflow for dose recalculations on independent electron density maps before the availability of an MR scanner dedicated to RT and also to fine tune the values for bulk density overrides. The second phase focussed on the clinical validation of sCT for treatment planning, definition of the exclusion criteria and quality assurance procedures. These corresponded, respectively, to phases nr. 1 and nr. 4 in the description provided by Moore-Palhares et al. [21]. In the preparatory phase, data from 30 randomly selected patients, distributed equally among non-stereotactic brain, male pelvis and female pelvis cases, treated between March 2022 and March 2023 using a conventional workflow, i.e. MR in diagnostic positioning, were analysed as a representative sample of the population treated in our department. The commissioning phase systematically analysed every patient that qualified for MR imaging since the MR scanner installation in April 2023. This led to the definition of exclusion criteria (Table 1) plus the inclusion of 30 patients in the current study. These had the same distribution as phase one and were treated with an MR-only planning workflow, i.e. all imaging in treatment position. In this phase, the sCT was utilized for dose calculation, although a CT was still acquired.

**Table 1:** Exclusion criterial table. This study included only non-stereotactic brain cases treated with a three point mask. Stereotactic cases are typically immobilized with a BrainLab, for higher accuracy, but are made of high-density material, which does not produce MR signal. Bone metastasis cases were excluded for two reasons: first, most palliative cases according to the SOP do not require MR examinations for contouring. Second, this study focused on target volumes located in soft tissue and the dosimetric accuracy of target volumes in the bones was beyond the scope.

**Condition**	**Reason for exclusion**	**Solution**
Patient requires RT-positioning material that is not MR-compatible.	MR images would not be possible.	Scan the patient in the MR in diagnostic position. Acquire a CT. Register in Eclipse MR and CT. Plan on CT.
PTV extends above L3 for pelvis and below C1 for brain.	sCT generation is not within the intended use.	Acquire MR and CT in treatment position. Plan on CT.
Diameter of the patient is too large and there are MR artefacts in the PTV area.	The skin contour is not reliable.	
Patient positioning is uncommon.	sCT generation not reliable with high pitch/roll.	
Brain post-op patients with open skull.	Open skull is reconstructed with closed skull in the sCT.	
Patient has hip implants or the PTV is only in the bone.	Beyond the scope of this study	Acquire MR and CT in treatment position. Plan on CT.

The study complied with the ethics protocol BASEC-Nr. 2023–01508 (cantonal ethics committee Zurich). All treatments were conducted using a C arm-Linac (Varian a Siemens Healthineers company, Erlangen, Germany) at the Department of Radiation Oncology of the University Hospital of Zurich and the prescribed doses are provided in the Supplementary Table 1. CT scans were performed with a Somatom Definition AS, equipped with carbon fiber radiotherapy couch, operated at 120 kVp. MR images were acquired with 3D distortion correction, either using multiple scanners within the radiology department (preparatory phase) or with a Siemens Sola 1.5 T with flat tabletop within the radiotherapy department (commissioning phase).

For each CT and MR scans in the commissioning cohort, patients were positioned supine with knee-fix immobilization device for pelvic treatments and a three-point mask (Civco Radiotherapy, Lowa, USA) for brain treatments to ensure comfort and reproducibility. Additionally, the MR setup required coils: a 30-channel flex coil fixed on arcs to avoid deformation of the body, and a 32-channel spine coil located under the flat tabletop for pelvic cases. For brain cases, two 18-channel flex coils are used.

The sCTs for dose calculation were generated sing the syngo RT Image Suite VB60 (Siemens Healthineers, Erlangen, Germany), a commercial software configured to create a calibration curve equivalent to a 120 kVp scan. The sCT generation was based on vendor-provided T1 VIBE Dixon sequences: brain scans were acquired in sagittal orientation with isotropic 1.50 mm voxels in 4:25 min, pelvis scans were acquired in transversal orientation with 1.50 mm voxels in-plane and 2 mm slice thickens in 4:12 min. Conversion from Hounsfield units (HU) values to ED was performed in the treatment planning system Eclipse v16.1 (Aria, Varian a Siemens Healthineers company, Erlangen, Germany), with the same HU look-up table used for CT scans.

Although Siemens imposes no strict exclusion criteria for utilizing sCT within the CE marking, except for generation limited to clinical datasets of the pelvis or brain, and appropriate patient's positioning for the specific anatomy, an institutional standard operating procedure (SOP) was developed, including a list of exclusion criteria based on clinical experience and literature to prevent predictable errors and avoid additional scans (Table 1) [22], [23], [24], [25].

### 2.2 Post-processing, contouring and planning

The digital imaging and communications in medicine (DICOM) nodes were configured at the scanners to facilitate the export of MR images and CT data to MIM v7.4.2 (MIM Software Inc, Cleveland, USA), which was used for automatic contour segmentation (contour ProtégéAI + auto-contouring v1.3.1) and linear-interpolation resampling of the sCT to 1x1x1 mm. The MR and sCT were imported in Eclipse with identical DICOM origins and identical frame of reference, therefore the images were intrinsically co-registered and no further action by the user was required.

In agreement with clinical workflow integration recommendations, axial T2 and axial T1 post-contrast sequences were utilized for contour refinement of the pelvis and brain, respectively [26]. The user origin was placed on MR-compatible fiducial markers for the brain and at the intersection of the umbilicus and the tabletop for the pelvis. Since pelvic volumes were treated tattoo-less, patient positioning was facilitated by Align RT (Vison RT Ltd., London, United Kingdom), a surface guided RT system. To identify the couch position, two MR-markers were placed on the tabletop and included in the MR field of view (FOV).

During planning, the T1 VIBE Dixon in-phase sequence was overlaid onto the sCT to ensure outer body contour correspondence. All patients were treated with VMAT plans created in Eclipse v16.1 (Aria) or 3D conformal plans for whole brain treatments. Plans were optimized and calculated on the sCT. The beam arrangement consisted of two to three coplanar 6 MV arcs or two lateral 6 MV fields. Each VMAT plan had jaw tracking activated, was normalized to target median and was calculated using Acuros v16.1 dose-to-medium algorithm with 1.5 mm grid resolution.

### 2.3 PSQA methods and automation

**Fig. 1:**
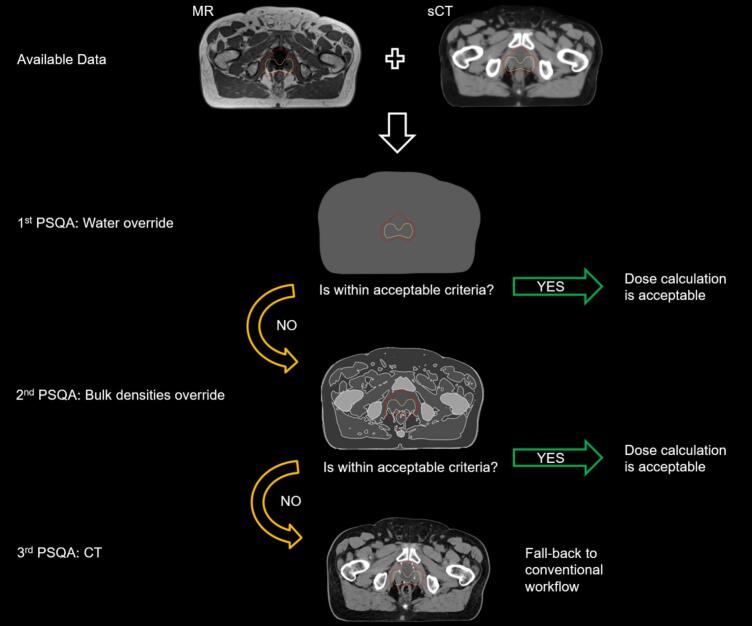
Overview of decision-making process of PSQA methods with independent ED maps of increasing complexity. PTV is in red and clinical target volume (CTV) in yellow. The patient nr. 15 from the commissioning phase is used for this illustration.

Three distinct methodologies to create separate ED maps and performing PSQA of sCT were explored, hereinafter referred to as: (A) uniform water override of the entire body mask defined on the MR, (B) bulk heterogeneous density override only for pelvic cases, and (C) CT only for patients included in the commissioning phase (Fig. 1).

Four tissue classes were manually contoured for each patient on the MR data in the axial slices of the PTV ± 2 cm. In the preparatory phase T1 or T2-weighted images with sufficient FOV and distortion correction were used, while in the commissioning phase this analysis was performed on the T1 VIBE Dixon in-phase. The tissue classes were:

- Bone defined as one tissue type including both cortical and trabecular parts
- Air delineated on the T2 image when available
- Fat contoured using a thresholding tool.
- Generic soft tissue as the remaining volume.

During the preparatory phase, average population values for bulk override HU were extrapolated and utilized instead of the ICRU 46 values [27] to improve dosimetric accuracy [28], [29]. Extensive evaluation of bone was conducted due to inconsistencies among previous studies [9], [27]. Bulk override was not performed for brain cases because it was challenging to clearly distinguish air cavities from bone on the MR sequences currently implemented, particularly in presence of artefacts [3], [18].

An in-house script was developed in C# with extra functionality for Eclipse scripting API (ESAPI) to automate the PSQA workflow through the following steps:

- Create a new structure set different from the one used in the clinical RTPlan.
- Rigidly copy the clinically approved plan on the new structure set while maintaining the original multi-leaf collimator openings.
- Override the structures of interest with defined HU values according to the PSQA.
- Recalculate the plan with the same setup and number of MU per beam.
- Analyze differences in percentage between the DVH values of the plan on the sCT and the PSQA, normalized by the prescribed dose.
- Create a PDF report (Supplementary Fig. 1).

### 2.4 Analysis

The evaluation was based on the ICRU-83 [30] recommended parameters for the PTV (Dmean, D98%, D95%, D2%), serial OAR (D2%) and parallel OAR (Dmean). A two-tailed z-test was performed in R Studio 4.2.0 for statistical analysis of each cohort, given the small sample sizes [31] and following related works [32]. The null hypothesis (H_0_) posited that the dose difference between the PTV Dmean values in PSQA and sCT equals 0 %.

The prerequisites of the z-test included a normal distribution of the population and known variance. Normal distribution for each cohort was assessed both visually and statistically via Shapiro-Wilk test, with H_0_ stating that the data in the sample follows a normal distribution [33]. The variance represents the significant deviation for clinical applications and was set to 1. The significance level p was established at 0.05, then corrected according to Bonferroni (m = 2) to 0.025 [34]. Data processing was performed using Python (PyCharm 2021.3).

## 3 Results

**Fig. 2:**
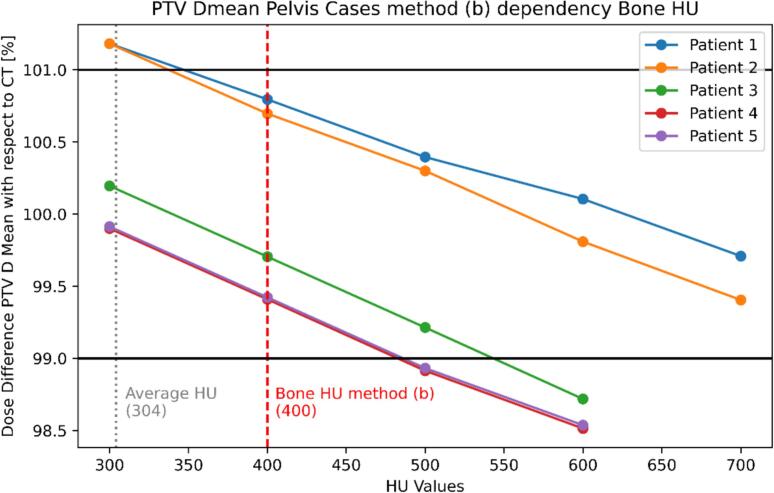
Dependency of the PTV Dmean dose difference on the bone HU values for method (B) across five pelvis cases. The red vertical dashed lines indicate the bulk HU utilized in method (B) (400 HU), the average HU measured for the five patients is in grey (304 HU). The black horizontal lines represent ± 1 % deviations in the PTV Dmean between the recalculated plans and the original plan.

An optimal value of 400 HU was identified for bone (Fig. 2), as PTV Dmean values were within ± 1 % for all patients and aligned with the ranges defined by ICRU 46 and other clinics [8].

**Fig. 3:**
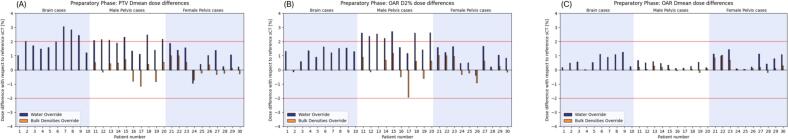
A-C: Relative signed deviation of the PTV Dmean (A), OAR D2% (B), OAR Dmean (C) calculated on ED maps obtained from the reference sCT against water override and bulk densities override divided by the prescription dose for the preparatory cohorts. OAR D2% corresponded to the highest near-maximum dose received by a serial OAR: brainstem for the brain cohort, rectum or bowel for male and female pelvis cohorts. OAR Dmean corresponded to Dmean received by a parallel OAR: brain-PTV for the brain cohort, bladder for male and female pelvis cohorts. The horizontal red lines indicate the limit of ± 2 %, while the vertical colour bands distinguish the patients in the three cohorts.

**Fig. 4:**
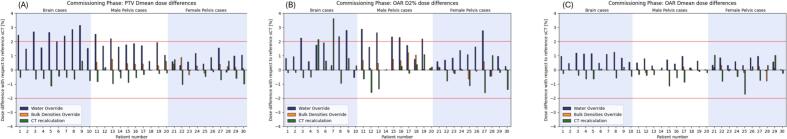
A-C: Relative signed deviation of the PTV Dmean (A), OAR D2% (B), OAR Dmean (C) calculated on ED maps obtained from the reference sCT against water override, bulk densities override and CT recalculation divided by the prescription dose for the commissioning cohorts. OAR D2% corresponded to the highest near-maximum dose received by a serial OAR: brainstem for the brain cohort, rectum or bowel for male and female pelvis cohorts. OAR Dmean corresponded to Dmean received by a parallel OAR: brain-PTV for the brain cohort, bladder for male and female pelvis cohorts. The horizontal red lines indicate the limit of ± 2 %, while the vertical colour bands distinguish the patients in the three cohorts.

As the complexity increased from (A)-(C), the DVH differences between the reference sCT and the PSQA method decreased. The relative dose differences for PTV Dmean, serial OAR D2%, and parallel OAR Dmean are presented for individual patients (Fig. 3, Fig. 4).

Generally, method (A) consistently showed higher dose values and no outliers exceeding 4 %. Method (B) demonstrated greater accuracy, was centered around zero with no outliers above 2 %. Method (C) also clustered around zero and remained within 1 % threshold, though some outliers were observed for OARs, especially D2%.

For PTV Dmean, method (A) stayed within 3 % for pelvis cohorts (mean < 2 %) and 4 % for brain cases (mean < 3 %), with standard deviations under 1 %. Method (B) remained a ± 2 % tolerance (mean < 1 %) and standard deviations within 1 %. Method (C) achieved the ± 1 % tolerance (mean < 1 %), except for brain patient 5, male pelvis case 12 and female pelvis case 22, where standard deviations slightly exceeded 1 %.

For OAR Dmean, method (B) showed a slightly positive shift, while all methods maintained average dose values and standard deviations below 1 %. For serial OAR, D2% mean values were similar to PTV Dmean across all methods: slightly above 2 % for (A), below 1 % for (B) and (C). In method (C), dose differences with respect to reference sCT were below 3 % for brain patient 5 and under 4 % for patient 7, still within dose tolerances for planning.

Supplementary Table 2-3 summarize the means and standard deviations of deviations for methods (A)-(C), patient cohorts and DVH points in the first and second phase. Additionally, box plots for the complete set of DVH points and the relative differences are reported (Supplementary Fig. 2, Fig. 3, Fig. 4).

**Table 2:** Overview of acceptance criteria for maximum observed deviations in PTV Dmean analysed during the preparatory phase. The deviations are reported for the combinations of PSQA methods (A) and (B) across three patient cohorts.

**Site**	**Water Override**	**Bulk Override**
Brain	PTV Dmean (−1%, +4%)	*Not investigated in the current study*
Male Pelvis	PTV Dmean (−1%, +4%)	PTV Dmean (−2%, +2%)
Female Pelvis	PTV Dmean (−1%, +4%)	PTV Dmean (−2%, +2%)

Supplementary Fig. 5 presents the p-values from the z-test for each cohort. PTV Dmean dose difference was statistically significant for (A) in every group. Instead, for (B) and (C) were not significantly different when evaluated. Therefore, the null hypothesis cannot be rejected, indicating that the population mean of these two PSQA methods was not significantly different from 0 ± 1 %.

## 4 Discussion

This work described the commissioning and implementation of PSQA for MR-only planning using a sCT software for clinical use in non-stereotactic brain, male pelvis and female pelvis patients. Various PSQA options were explored.

Our analysis involved 60 cases equally distributed among brain non-stereotactic, male pelvis and female pelvis patients. The preparatory phase (N = 30) established bulk density HU values and defined PSQA acceptance criteria for approving sCT dose (Table 2), which were validated during the commissioning phase. The preparatory phase employed MR data from the radiology department before the installation of a scanner in the radiotherapy department. This method is proposed to gain experience upfront and have a faster commissioning phase once a dedicated scanner becomes available. This approach can be implemented by other institutions planning to commission sCT on a radiotherapy dedicated scanner. All patients in the commissioning phase (N = 30) were successfully treated with plans calculated on sCT.

The HU values assigned for air, fat, soft tissue and bone in method (B) were HU −1000, −75 [32], 7 [32] and 400, respectively. The PTV Dmean was chosen as reference value for DVH comparison. On average, the PTV Dmean deviations met the clinical applicability requirement of 2 % [19], therefore it was judged as applicable for clinical treatments the use sCT for dose calculation. One limitation of this study is the focus on PTV Dmean as the main parameter. This is motivated by the proposal of a PSQA method that should be swift and easy to implement, such that it can be performed for every single patient, along with the tight correlation of PTV Dmean to the MU normalization of radiotherapy plans. Further PTV DVH parameters such as D2% and D98% were also analyzed and found to be in good correlation with Dmean, thus supporting the choice of Dmean as the surrogate for a swift PSQA method (Supplementary Fig. 6).

The Swiss society for radiobiology and medical physics recommendations No.15, recommends PSQA for each intensity-modulated plan prior to treatment. This includes an independent check calculation of monitor units (MU), ensuring dose tolerances within ± 3–5 % [35]. While the original aim of the secondary MU check was intended to verify the dose calculation engine, replacing a CT with sCT has also an influence in MU calculation and its contribution should fall within this uncertainty budget.

The greatest discrepancy in DVH results was observed for PTV D2%. Dose calculation differences for PTV Dmean using method (C) were minimal (range: −1.1 % to 0.7 %). Deviations slightly over 1 % in male pelvis patients 11, 13 and 15 were linked to significant changes in body outline on the MR image compared to the CT image (Supplementary Fig. 7).

QA dose recalculation relies on the body contour, and any discrepancies, arising from factors like breathing or motion artefacts could potentially introduce bias into the recalculation results.

In the commissioning phase, differences in positioning between MR and CT scans were noted for female pelvis patients number 22 and 30, and male pelvis patient 12. However, for the latter, the comparison of HU profiles between sCT and CT images revealed consistent total attenuation of the radiation in the volume, validating the accuracy of the sCT for dose calculation. To ensure adequate dose distribution, a deformable registration between CT and T1 VIBE Dixon was performed, followed by dose recalculations on the deformed CT with preset MU, resulting in a reduction in the deviation in PTV Dmean from −1.6 % to 0.2 % (Supplementary Fig. 8). For all the other cases, only a rigid registration sCT-CT was performed and the dosimetric results in the commissioning phase (Fig. 4) supported this approach showing that the anatomical differences sCT-CT have a limited impact. These, summed to the sCT-CT HU differences, led to dose deviations always within 2 %. An exemplary case of rigid registration is provided in Supplementary Fig. 9.

The calculations for brain patient number seven showed a deviation above 3 % (but < 4 %) due to the PTV's location in paranasal cavities, which requires further investigation. No OAR were overdosed in any calculations.

Water override recalculations showed a significant difference, while bulk override and CT recalculations aligned with the reference mean. This suggests that bulk override and CT recalculations do not lead to significant deviations, unlike water override. It was concluded that easily implementable recalculation methods, such as (A), may be employed to identify outliers with large deviations (+4%/-1%) and should be performed as PSQA for every patient. Method (B), though more robust, is also time-consuming; therefore, we propose performing it when (A) fails and monthly as QA check for the software, in accordance with ESTRO guidelines [36]. (B) consistently resulted in average deviations within ± 2 % of the prescribed dose at ICRU reference points and DVH parameters, meeting the clinical applicability requirement of 2 % [19]. Therefore, if method (A) falls outside limits, we propose to perform method (B) as PSQA, with method (C) as fall-back if both (A) and (B) fail (Fig. 1).

MR-only planning for non-stereotactic brain, male pelvis and female pelvis has been clinically implemented at the University Hospital of Zurich with prospective PSQA methods to ensure patient-safe workflow.

In the commissioning phase, limitations were observed, particularly in brain cases where sCT resolution was reduced due to memory constraints, hindering the use of Rapid Plan models for plan optimization. Some limitations related to sCT generation were successfully handled within MR-only planning workflow, as detailed in Supplementary Figs. 10–16 and described in the exclusions criteria table (Table 1), with the aim to minimize the number of patients needing to switch to the conventional workflow.

Manual review of the MR sequences and reconstructed sCT data are recommended [37] and were performed it in the current study through visual inspection while the patient was still in the scanner. Generating the sCT while scanning diagnostic sequences are performed allows to identify unexpected artefacts or missed exclusion criteria and gives the opportunity to re-scan the T1 VIBE Dixon at the end of the protocol. This process is more likely to ensure successful sCT generation.

Finally, this study focused solely on dose calculation QA for sCT. While sCT can be directly used for cone-beam CT matching or to generate digitally reconstructed radiographs (DRRs) for kV-kV matching, it may introduce new uncertainties into the RT workflow [18], requiring dedicated image guided RT (IGRT) testing. IGRT testing was evaluated separately and is outside the scope of this manuscript.

We have proposed a reliable PSQA framework for MR-only planning. Method (A) effectively identifies potential large outliers (−1%/+4%), while method (B) enhances detection to the 2 % level, in agreement with sCT clinical applicability requirements. (C) may be considered if previous PSQA methods fail. The defined acceptance criteria could serve as a powerful tool toward standardizing MR-only planning and facilitating clinical implementations.

Further development of auto-segmentation of MR-based OAR with CNNs [38], could reduce workloads in RT department and may be essential for widespread of an MR-only planning workflow.

## Declaration of competing interest

The authors declare that they have no known competing financial interests or personal relationships that could have appeared to influence the work reported in this paper.
