# Supplementary material for: Clinical implementation of patient-specific quality assurance for synthetic computed tomography
Source: Phys Imaging Radiat Oncol. 2025 Apr 4;34:100764. doi: 10.1016/j.phro.2025.100764 (PMC12005318; doi:10.1016/j.phro.2025.100764)
Supplement: Supplementary Data 1 [file mmc1.docx]

**Supplementary materials**

Supplementary Table 1. Dose prescriptions of the 60 patients included in this study.

| Body site | Patient number | Dose prescription for patient in preparatory phase | Dose prescription for patient in commissioning phase |
| --- | --- | --- | --- |
| Brain | 1 | 10 x 2.00 Gy | 30 x 2.00 Gy |
|  | 2 | 15 x 2.67 Gy | 30 x 2.00 Gy |
|  | 3 | 15 x 2.67 Gy | 30 x 2.00 Gy |
|  | 4 | 20 x 2.00 Gy | 10 x 2.50 Gy |
|  | 5 | 30 x 2.00 Gy | 33 x 1.80 Gy |
|  | 6 | 33 x 1.80 Gy | 3 x 3.00 Gy |
|  | 7 | 30 x 2.00 Gy | 28 x 1.80 Gy |
|  | 8 | 30 x 2.00 Gy | 30 x 2.00 Gy |
|  | 9 | 28 x 1.80 Gy | 15 x 2.67 Gy |
|  | 10 | 30 x 2.00 Gy | 33 x 1.80 Gy |
| Pelvis male | 11 | 30 x 2.00 Gy | 30 x 2.00 Gy |
|  | 12 | 5 x 2.31 Gy | 30 x 2.00 Gy |
|  | 13 | 20 x 3.00 Gy | 30 x 2.00 Gy |
|  | 14 | 33 x 2.31 Gy | 5 x 7.00 Gy |
|  | 15 | 28 x 2.00 Gy | 28 x 2.00 Gy |
|  | 16 | 20 x 3.00 Gy | 28 x 2.00 Gy |
|  | 17 | 20 x 3.00 Gy | 5 x 5.00 Gy |
|  | 18 | 20 x 3.00 Gy | 5 x 5.00 Gy |
|  | 19 | 20 x 3.00 Gy | 28 x 2.00 Gy |
|  | 20 | 3 x 2.00 Gy | 20 x 2.00 Gy |
| Pelvis female | 21 | 3 x 1.80 Gy | 25 x 2.30 Gy |
|  | 22 | 27 x 1.80 Gy | 25 x 2.30 Gy |
|  | 23 | 5 x 4.00 Gy | 25 x 1.80 Gy |
|  | 24 | 25 x 1.80 Gy | 16 x 2.50 Gy |
|  | 25 | 13 x 3.00 Gy | 27 x 1.80 Gy |
|  | 26 | 25 x 2.20 Gy | 25 x 1.80 Gy |
|  | 27 | 25 x 1.80 Gy | 28 x 1.80 Gy |
|  | 28 | 3 x 1.90 Gy | 25 x 1.80 Gy |
|  | 29 | 3 x 1.80 Gy | 28 x 1.80 Gy |
|  | 30 | 20 x 1.80 Gy | 25 x 2.30 Gy |

Supplementary Table 2. Quantitative values of the dosimetric differences for the DVH dosimetric points between the calculations performed on the reference sCT against the methods (A) and (B) in the preparatory phase.

| **Parameter** | **QA approach** | **Brain cases**  **Mean ± Standard Deviation [%]** | **Male Pelvis cases**  **Mean ± Standard Deviation [%]** | **Female Pelvis cases**  **Mean ± Standard Deviation [%]** |
| --- | --- | --- | --- | --- |
| PTV Dmean | Water | 1,94 ± 0,67 | 1,90 ± 0,46 | 0,83 ± 0,84 |
|  | Bulk densities | / | 0,02 ± 0,70 | 0,13 ± 0,59 |
| PTV D2% | Water | 2,37 ± 0,61 | 1,97 ± 0,47 | 0,82 ± 0,81 |
|  | Bulk densities | / | 0,45 ± 0,60 | 0,41 ± 0,94 |
| PTV V95% | Water | 2,1 ± 0,77 | 1,74 ± 0,49 | 0,77 ± 0,79 |
|  | Bulk densities | / | -0,74 ± 2,04 | -0,2 ± 0,25 |
| PTV D98% | Water | 2,04 ± 0,70 | 1,65 ± 0,46 | 0,81 ± 0,81 |
|  | Bulk densities | / | -1,50 ± 4,17 | -0,8 ± 1,18 |
| OAR D2% | Water | 1,13 ± 0,54 | 2,19 ± 0,57 | 0,88 ± 0,68 |
|  | Bulk densities | / | 0,08 ± 0,92 | 0,23 ± 0,66 |
| OAR Dmean | Water | 0,64 ± 0,43 | 0,38 ± 0,21 | 0,74 ± 0,50 |
|  | Bulk densities | / | 0,07 ± 0,14 | 0,33 ± 0,40 |

Supplementary Table 3. Quantitative values of the dosimetric differences for the DVH dosimetric points between the calculations performed on the reference sCT against the methods (A)-(C) in the commissioning phase.

| **Parameter** | **QA approach** | **Brain cases**  **Mean ± Standard Deviation [%]** | **Male Pelvis cases**  **Mean ± Standard Deviation [%]** | **Female Pelvis cases**  **Mean ± Standard Deviation [%]** |
| --- | --- | --- | --- | --- |
| PTV Dmean | Water | 2,28 ± 0,57 | 1,70 ± 0,51 | 0,74 ± 0,47 |
|  | Bulk densities | / | 0,34 ± 0,27 | 0,20 ± 0,33 |
|  | CT | -0,46 ± 0,48 | -0,44 ± 0,36 | -0,26 ± 0,58 |
| PTV D2% | Water | 2,70 ± 0,58 | 2,06 ± 0,60 | 0,82 ± 0,51 |
|  | Bulk densities | / | 0,51 ± 0,37 | 0,35 ± 0,43 |
|  | CT | -0,26 ± 0,49 | -0,39 ± 0,59 | -0,19 ± 0,81 |
| PTV V95% | Water | 2,33 ± 0,47 | 1,56 ± 0,49 | 0,73 ± 0,48 |
|  | Bulk densities | / | 0,05 ± 0,38 | 0,08 ± 0,29 |
|  | CT | -0,70 ± 0,63 | -0,82 ± 0,41 | -0,33 ± 0,77 |
| PTV D98% | Water | 2,21 ± 0,47 | 1,54 ± 0,56 | 0,65 ± 0,48 |
|  | Bulk densities | / | -0,07 ± 0,52 | 0,00 ± 0,32 |
|  | CT | -0,97 ± 1,00 | -0,93 ± 0,64 | -0,30 ± 0,96 |
| OAR D2% | Water | 1,32 ± 1,00 | 1,65 ± 0,98 | 0,98 ± 0,81 |
|  | Bulk densities | / | 0,51 ± 0,41 | -0,03 ± 0,32 |
|  | CT | 0,52 ± 1,41 | -0,18 ± 0,77 | -0,42 ± 0,76 |
| OAR Dmean | Water | 0,92 ± 0,30 | 0,45 ± 0,31 | 0,48 ± 0,33 |
|  | Bulk densities | / | 0,02 ± 0,10 | -0,02 ± 0,29 |
|  | CT | -0,27 ± 0,31 | -0,37 ± 0,37 | -0,12 ± 0,81 |


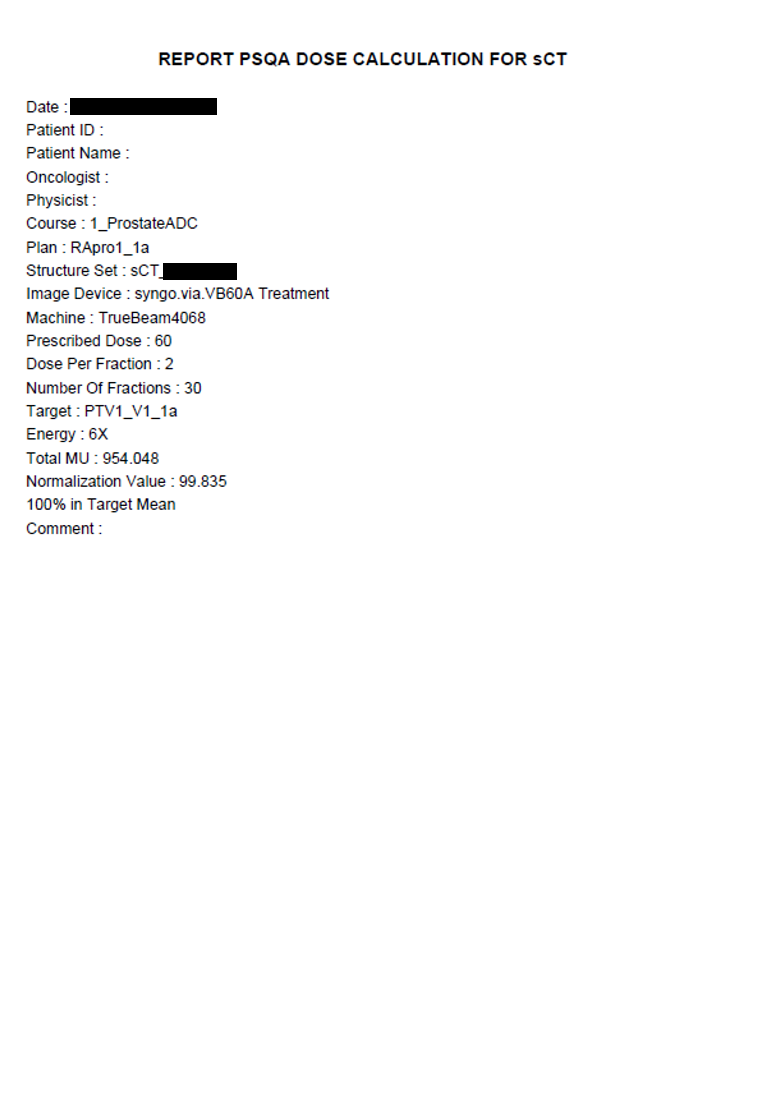


Supplementary Figure 1. Example first page of a PDF report of PSQA with information regarding the original plan on sCT.


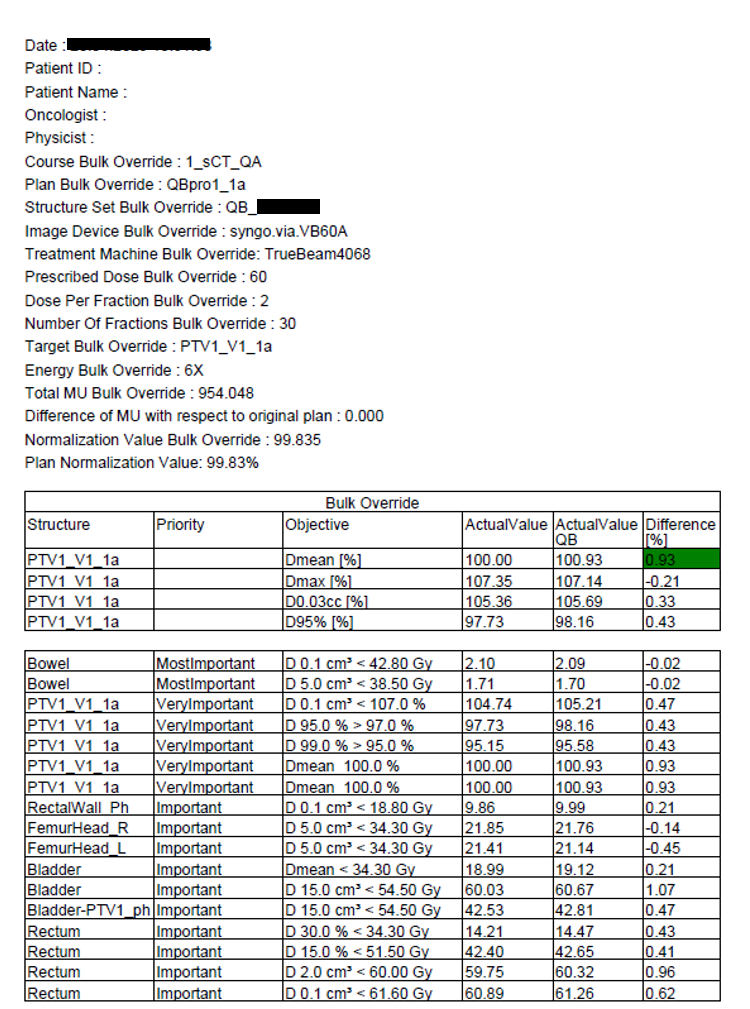


Supplementary Figure 1-continued. Example second page of PDF report of PSQA with information regarding the recalculated plan with bulk override.

1.
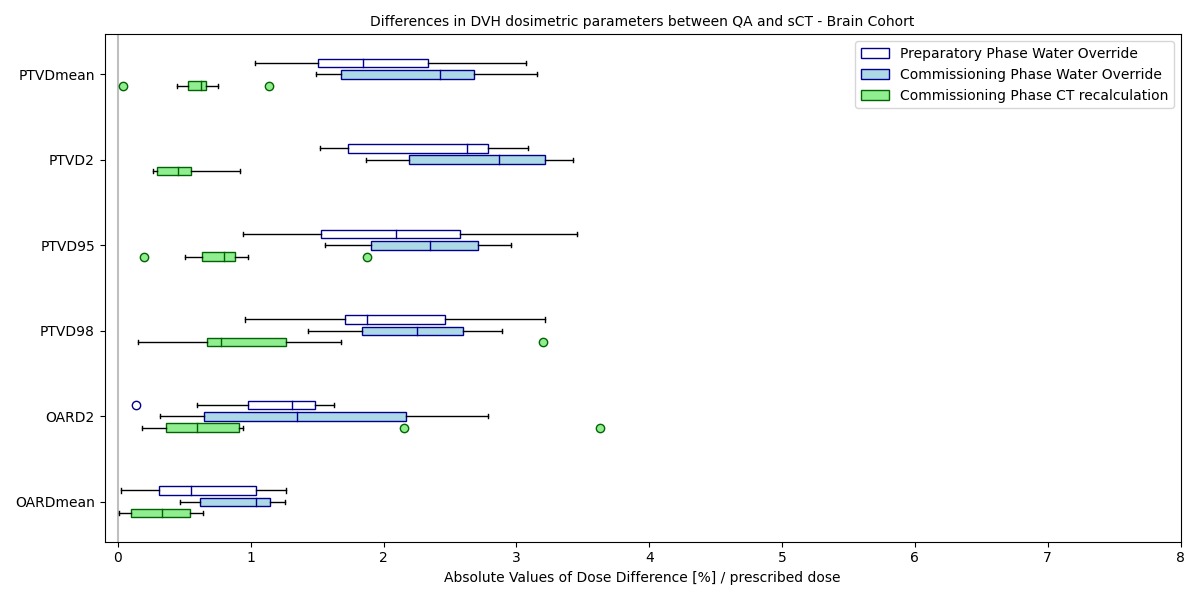

2.
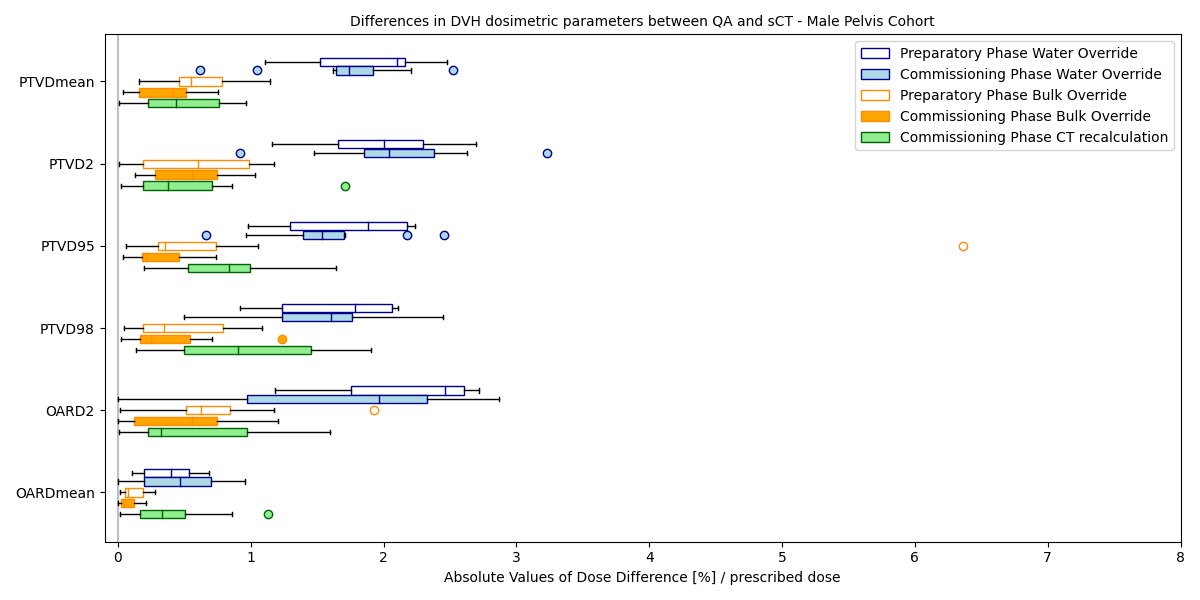

3.
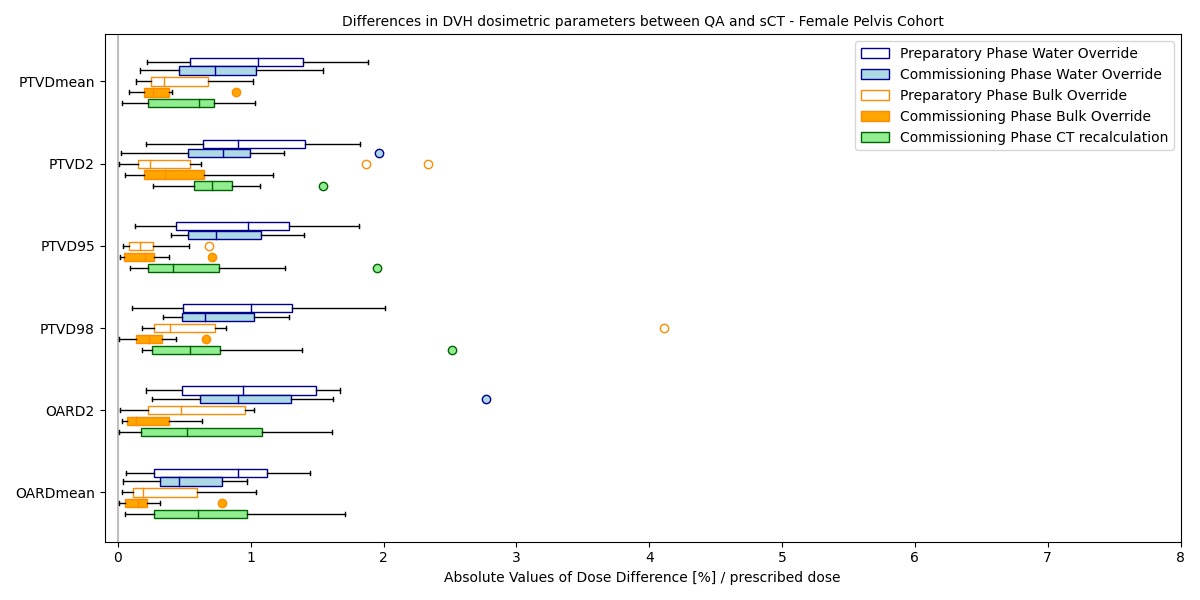


Supplementary Figure 2. A-C: Boxplots of the absolute values of the relative differences for the DVH dosimetric points calculated on sCT against water override, bulk densities override and CT recalculation for the brain cohort (A), male pelvis cohort (B), female pelvis cohort (C).

1.
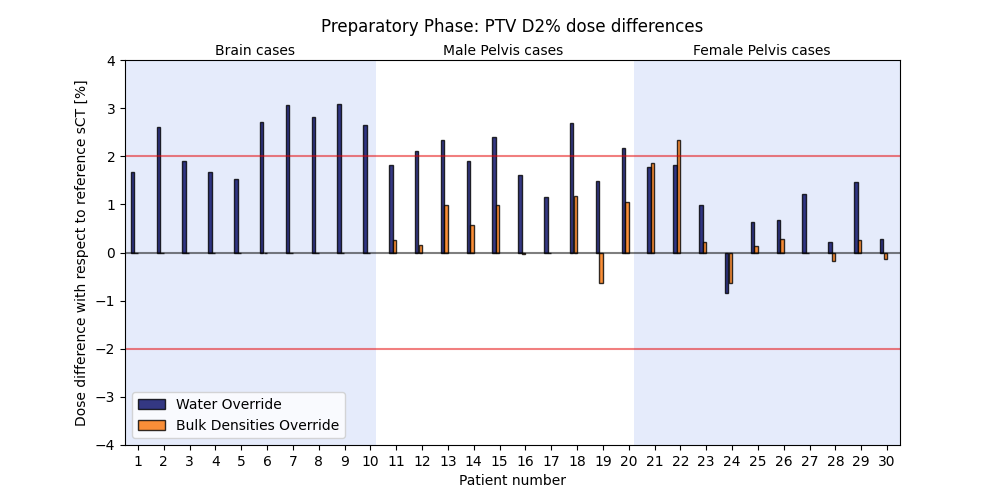

2.
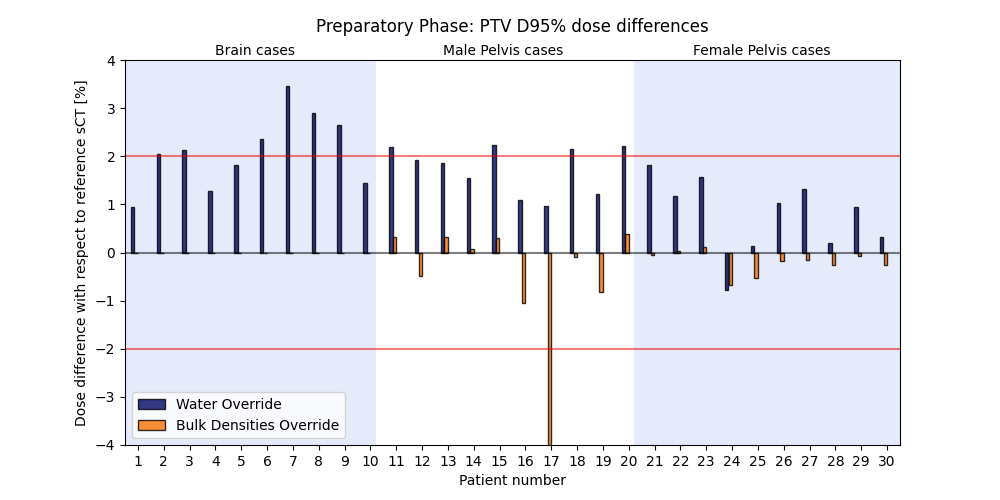

3.
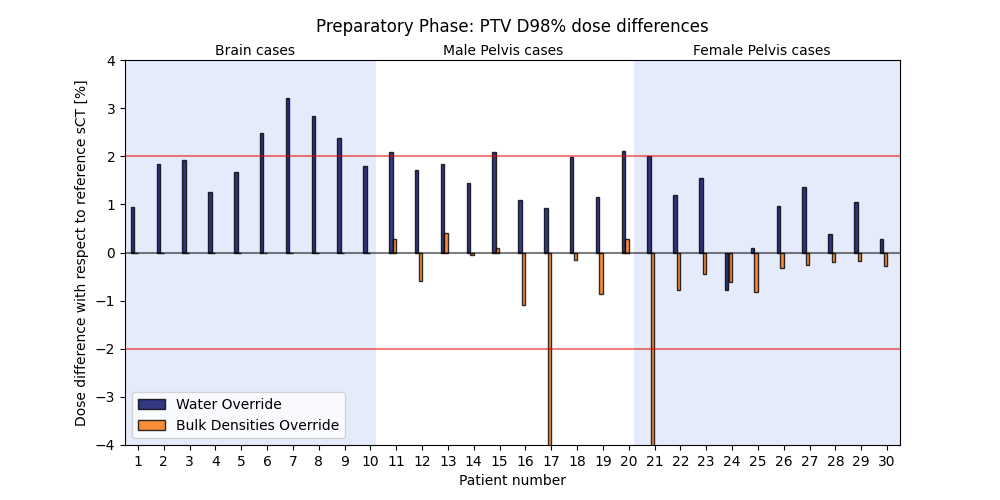


Supplementary Figure 3. A-C: Relative signed deviation of the PTV D2% (A), PTV D95% (B) and PTV D98% (C) calculated on ED maps obtained from the reference sCT against water override, bulk densities override for the preparatory cohorts. The horizontal red lines indicate the limit of ±2%, while the vertical colour bands distinguish the patients in the three cohorts.

1.
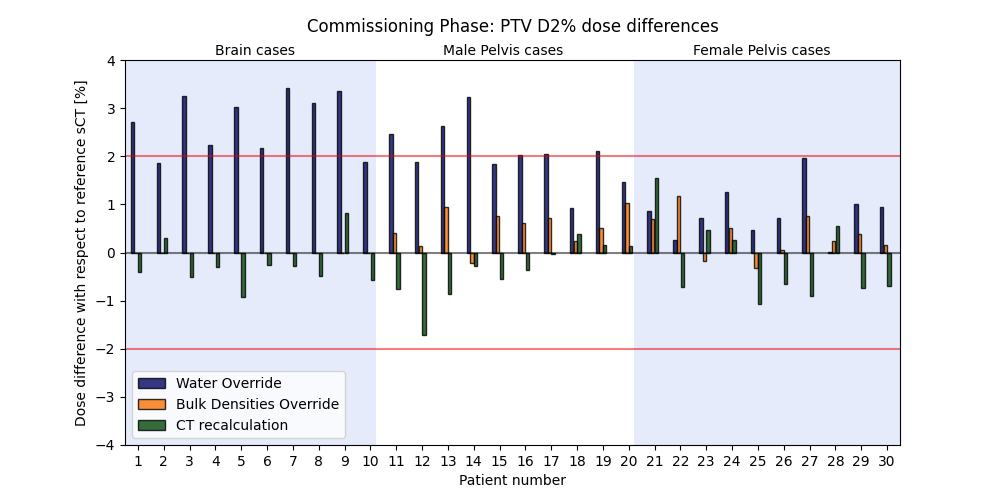

2.
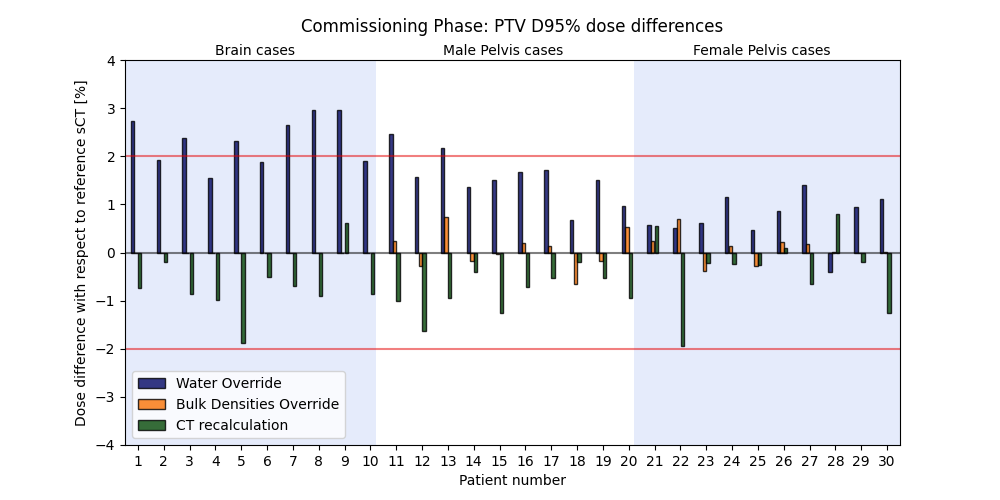

3.
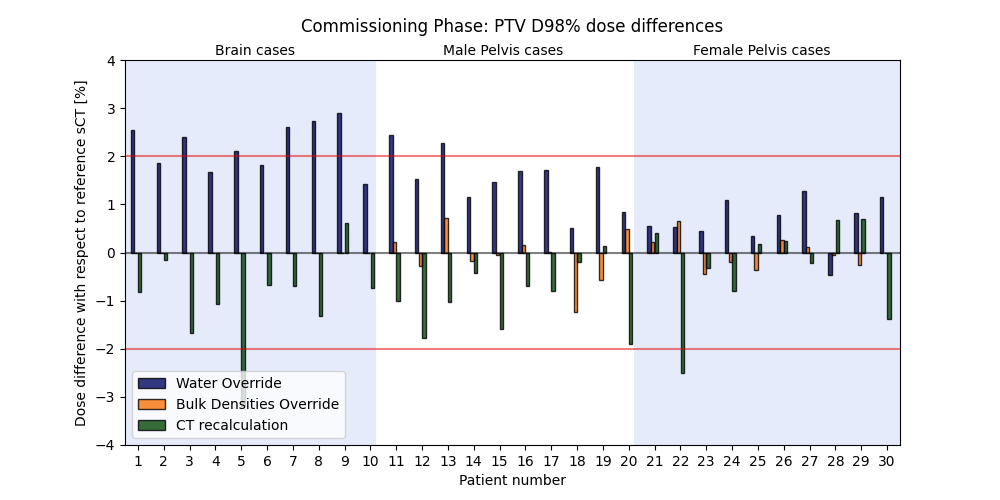


Supplementary Figure 4. A-C: Relative signed deviation of the PTV D2% (A), PTV D95% (B) and PTV D98% (C) calculated on ED maps obtained from the reference sCT against water override, bulk densities override and CT recalculation for the commissioning cohorts. The horizontal red lines indicate the limit of ±2%, while the vertical colour bands distinguish the patients in the three cohorts.

**
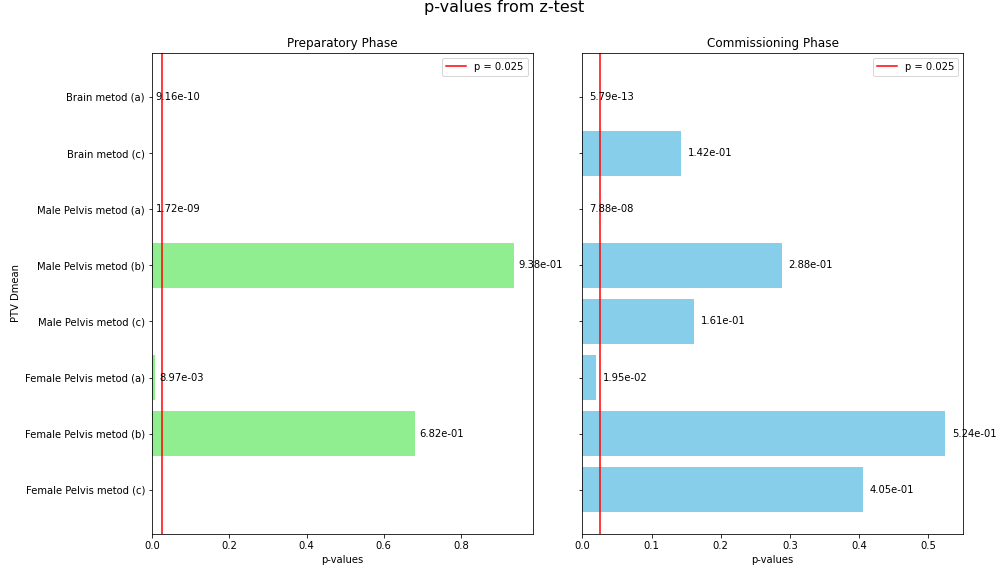
**

Supplementary Figure 5: The p-values from the z-test for each cohort are reported both for the preparatory (left) and for the commissioning phase (right). The percentage PTV Dmean dose difference was statistically significant for water override recalculations across all treatment sites (p value < 0.025). Instead, for bulk override and CT recalculations when evaluated, resulted not significantly different (p value > 0.025). The red vertical line represents p-value = 0.025, which refers to a 0.05 threshold corrected by Bonferroni (m=2).


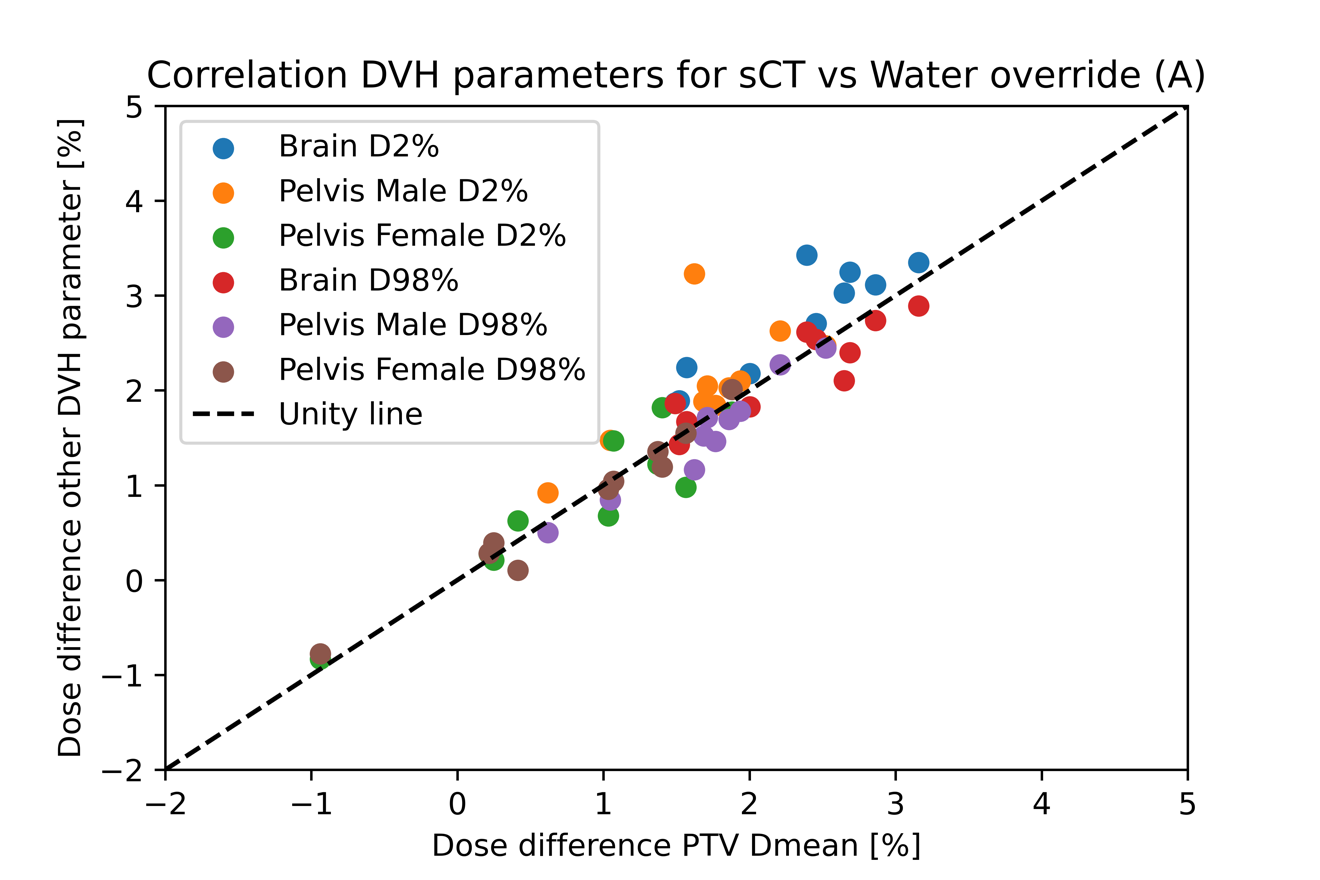


Supplementary Figure 6. Correlation of PTV Dmean with PTV D2% (r = 0.86) and PTV D98% (r = 0.95) for the patients in the commissioning phase.


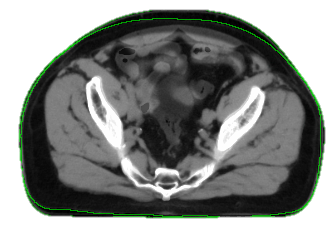

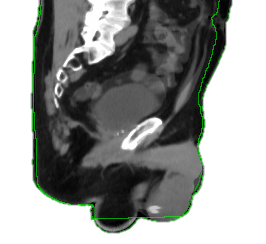


Supplementary Figure 7: An exemplary case of a patient with different body outline in sCT compared to CT. The bladder appears fuller on the CT, resulting in an anterior change of the body contour. The body contour with a smaller outline refers to the sCT, while the one with a larger outline refers to the CT. Patient nr. 13 of the commissioning phase. Windowing of the sCT and CT -125 HU/225 HU.


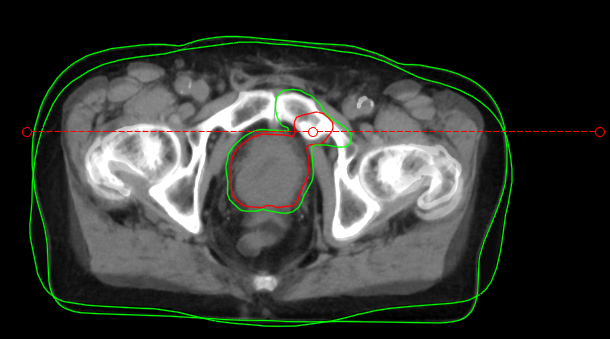

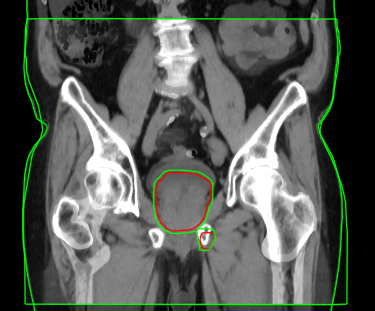

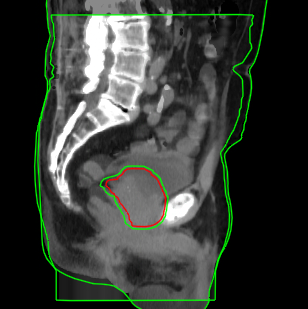


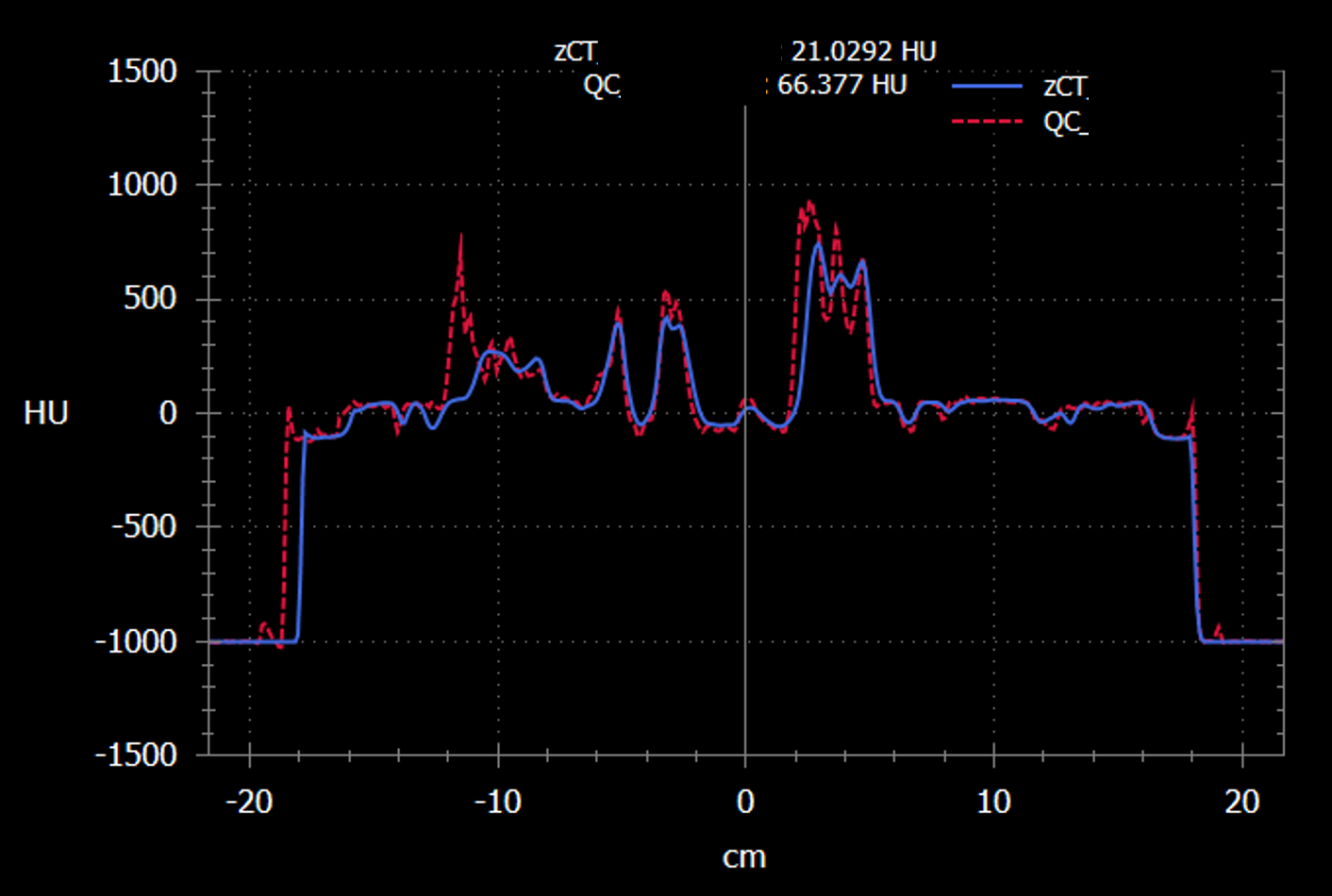


Supplementary Figure 8. An exemplary case of patient with different position between CT and sCT. The PTV high dose level (red) and PTV low dose level (green) contours are also displayed. In the sCT, the patient appears more relaxed and adherent more to the couch. Whilst in the CT the patient is tense, leading to muscle contraction and lack of complete adherence to the couch. This significant change in position leads to a PTV Dmean deviation larger than 1%, when recalculating on the CT. A qualitative comparison of the general appearance of HU in sCT and CT images was performed using HU profiles in the TPS. The HU of sCT and CT were consistent including the ones in the symphysis. When comparing HU profiles it has to be taken into consideration that the HU profiles in CT images are characterized by higher and thinner peaks, because CT images have higher resolution compared to the sCT. The sCT native voxel size is larger compared to CT images, this leads to volume averaging which is visualized as more blurred peaks (lower and broader). The relative dose difference between the reference sCT and the CT are reported for the axial slice. Patient nr. 12 of the commissioning phase. Windowing of the sCT and CT -125 HU/225 HU.


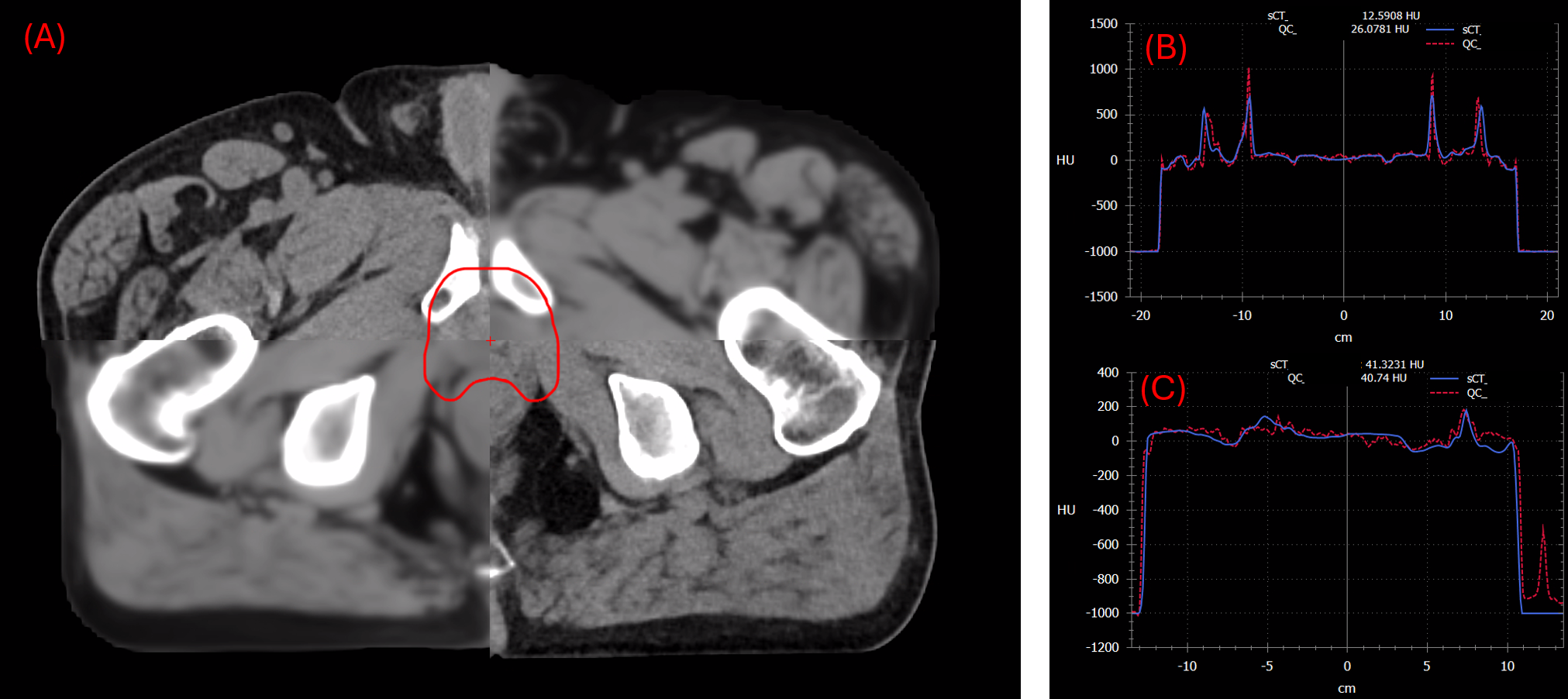


Supplementary Figure 9. Exemplary case of rigid registration between sCT and CT. The axial slice (A) shows the isocenter used in by the treatment fields and the PTV contour in red. The profiles (B) and (C) were drawn passing by the isocenter and show the voxel values of the sCT and CT along the directions left-right and anterior-posterior, respectively. Patient nr. 19 of the commissioning phase. Windowing of the sCT and CT -125 HU/225 HU.


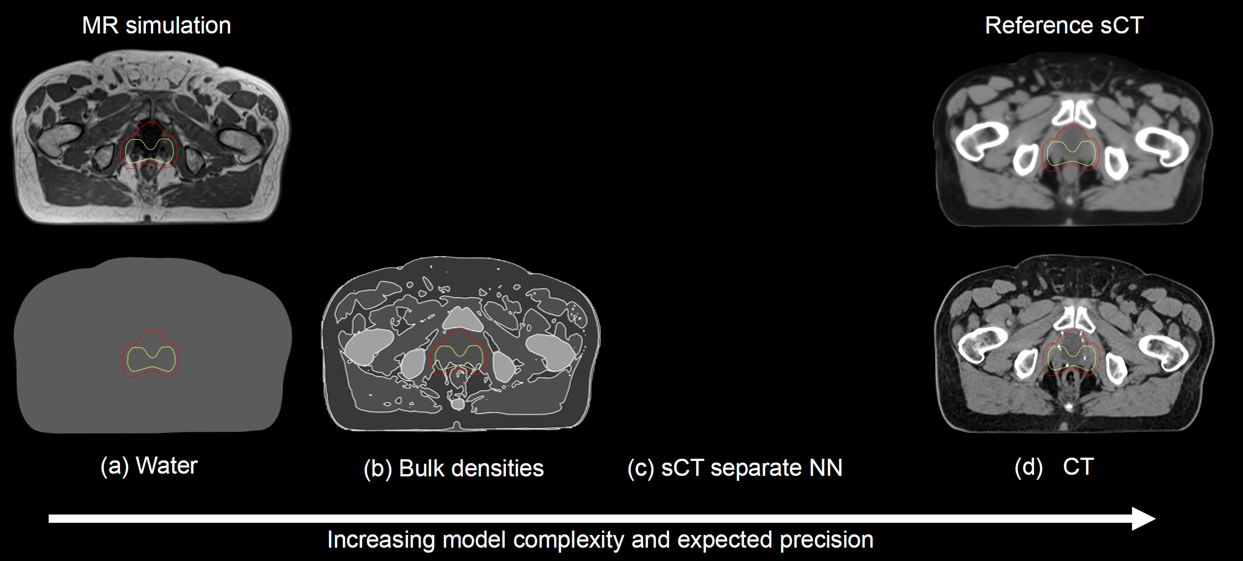

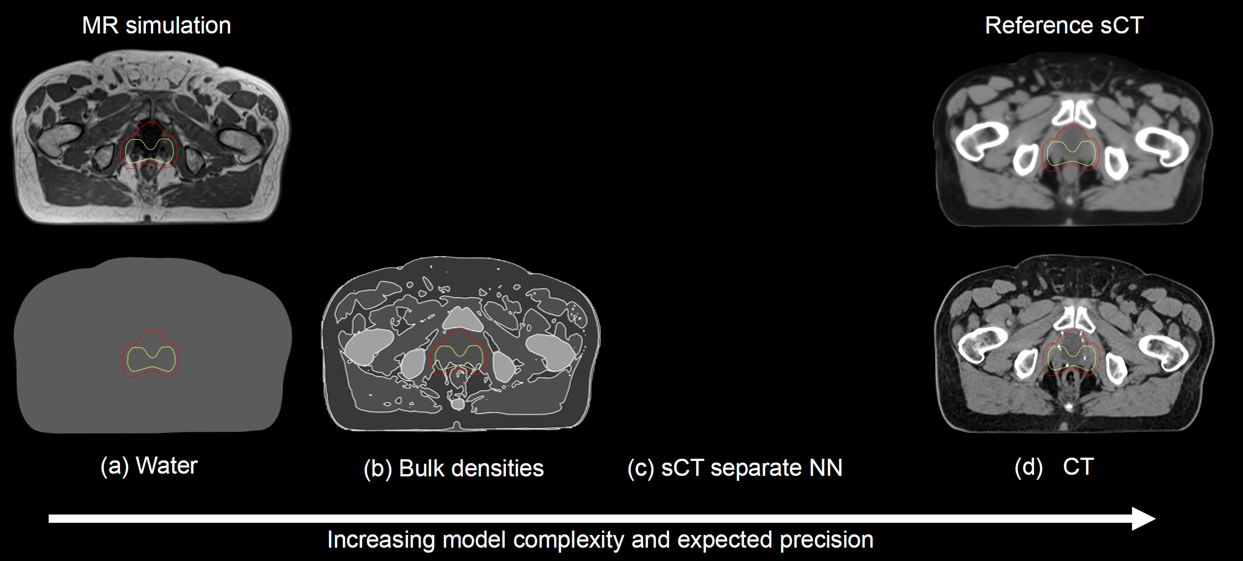

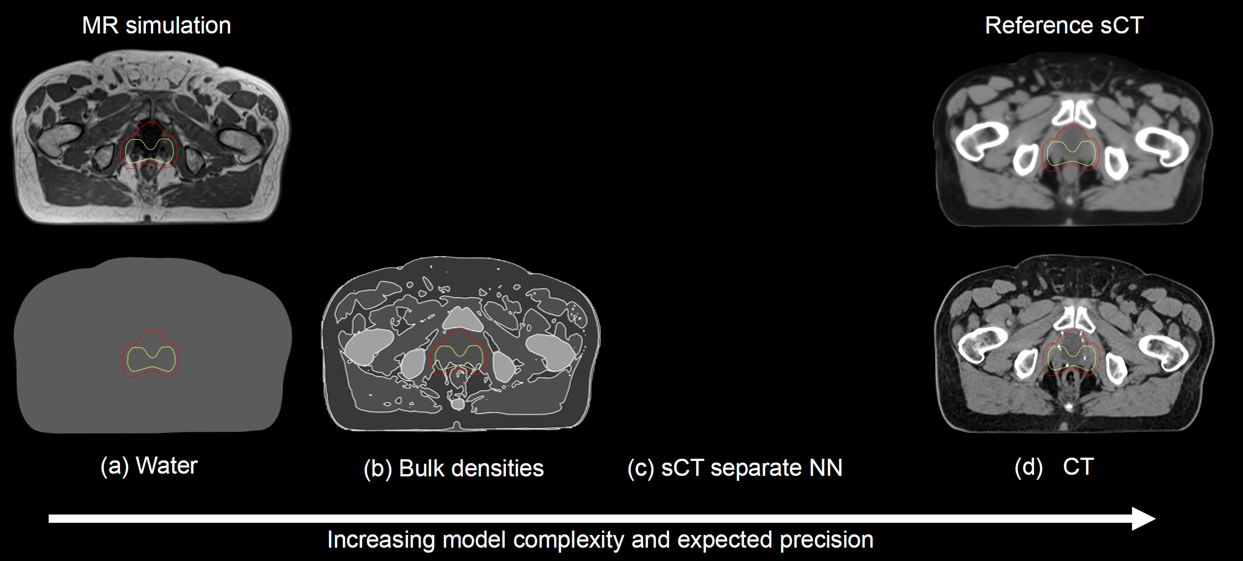


c)

b)

a)

Supplementary Figure 10. Example of patient for salvage treatment with surgical clips in prostate lodge. PTV (in red) and CTV (in yellow) are highlighted. Clips can be clearly visualized as hyperdense in CT (a), but are not detected in MR sequences (b) and as a consequence absent in sCT (c). Patient nr. 15 of the commissioning phase. Windowing of the sCT and CT -125 HU/225 HU.


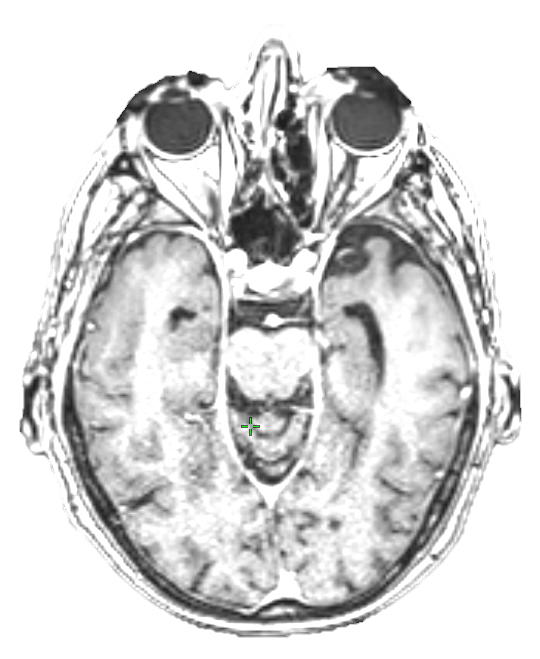

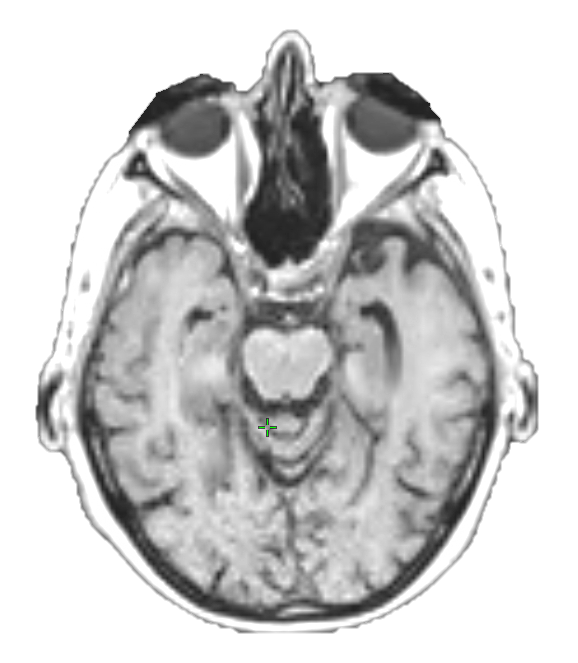

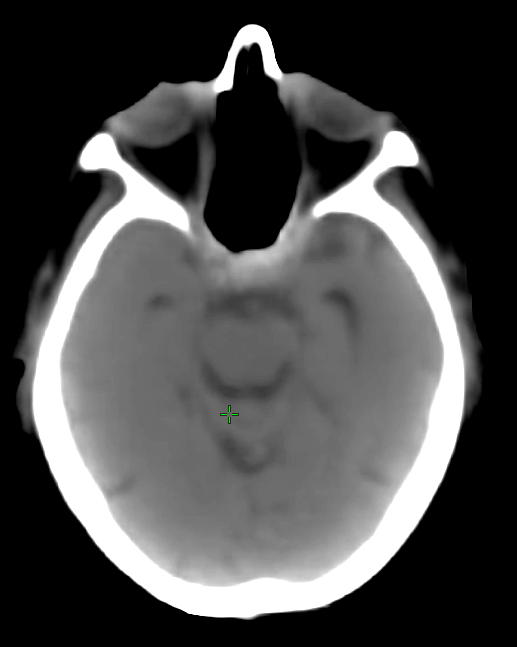


Supplementary Figure 11. Exemplary case of artefact present in the T1 VIBE Dixon sequence (middle) affecting the reconstruction of the eyes in the sCT (right). The eyes of the patient are better visualized on the T1 sequence (left). Contouring was unproblematic as on the T1 sequence the eyes were correctly displayed. This case was treated with standard beam arrangement: two lateral fields (273° and 87°) with multi leaf collimator (MLC) closed on the eyes. Therefore, the beam is not passing through the eyes and the artefact is not relevant for dose calculation. Patient nr. 4 of the commissioning phase. Windowing of the sCT -20 HU/100 HU.


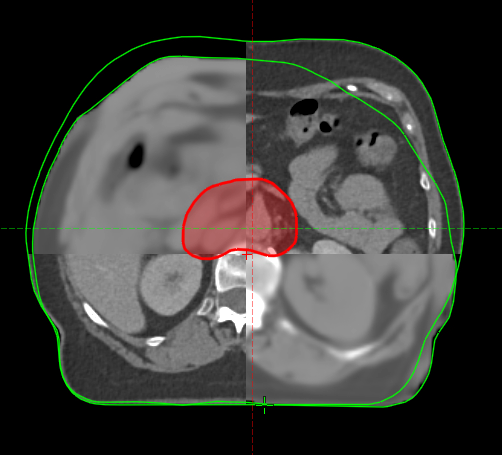

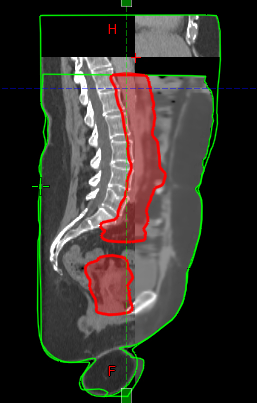

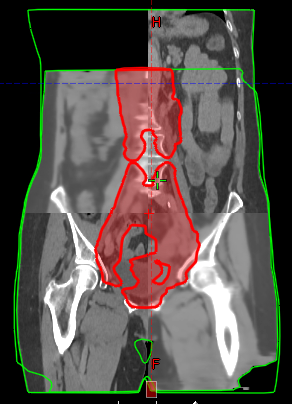


Supplementary Figure 12. Example of gynecological PTV (in red) extending to first lumbar spine. CT and sCT are blended, with both body outlines highlighted in green. The PTV extends beyond the sCT FOV and in the most cranial slices the body outline of the patient presents artefacts. Patient not included in the preparatory nor in the commissioning phase due to the extension of the PTV up to L1. Windowing of the sCT and CT -125 HU/225 HU.


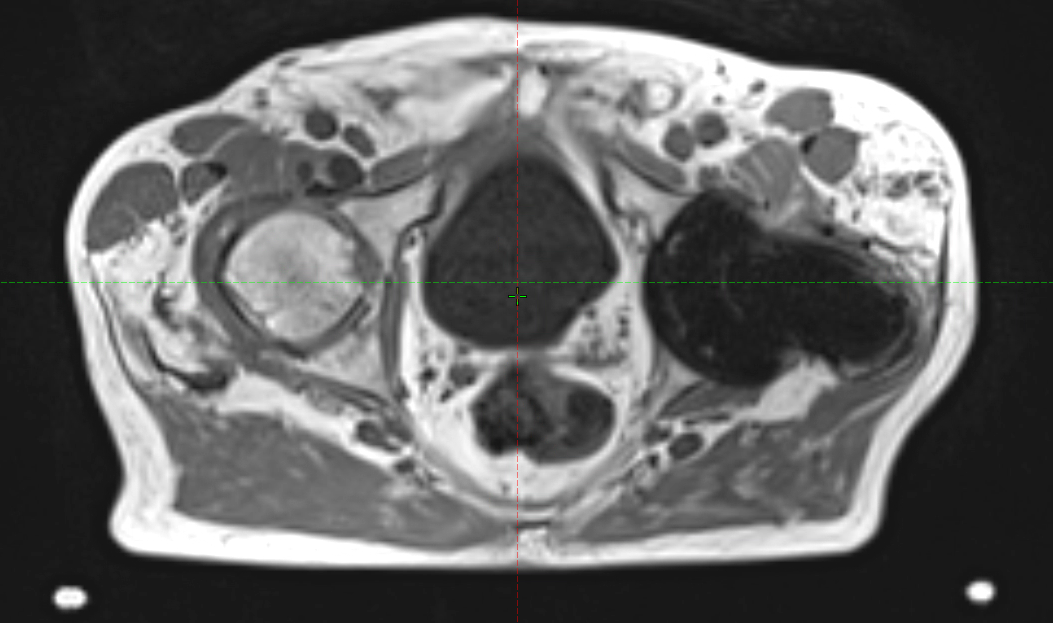


Supplementary Figure 13. Example prosthesis in Dixon in-phase sequence. Patient not included in the preparatory nor in the commissioning phase due to the hip implant.


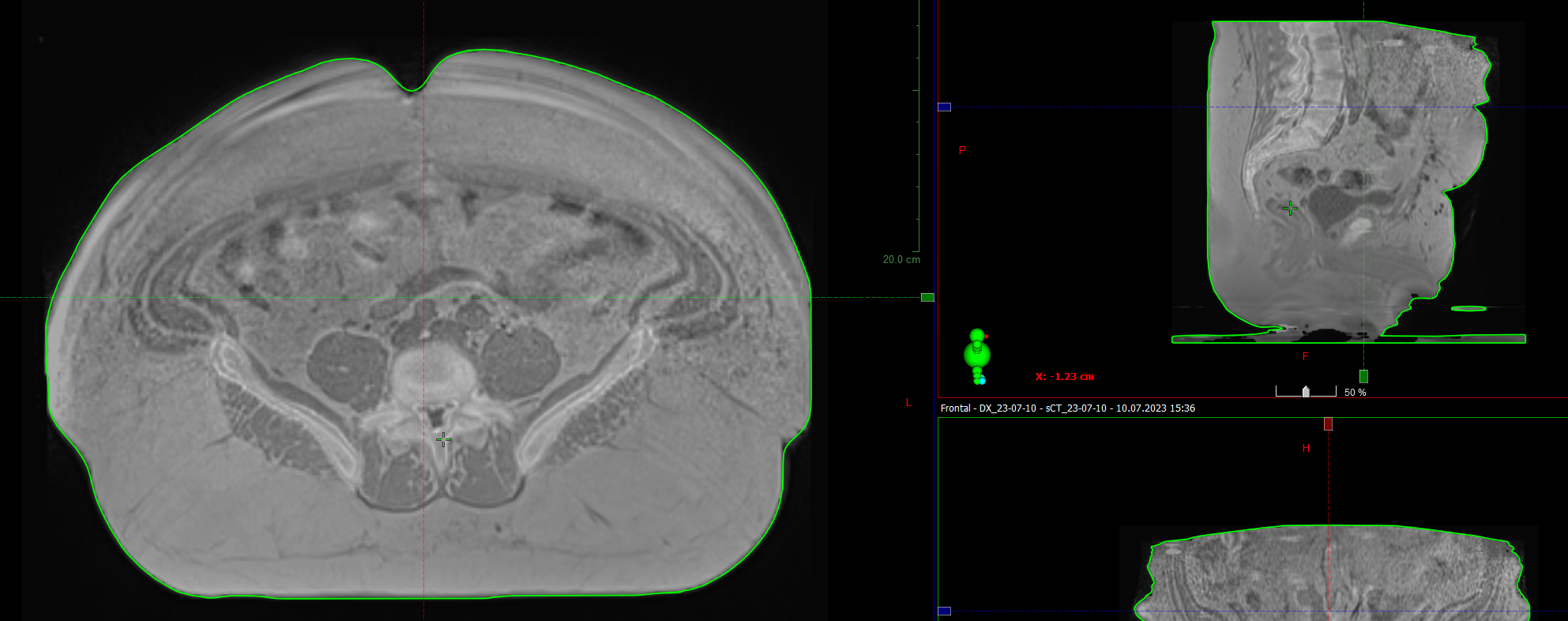

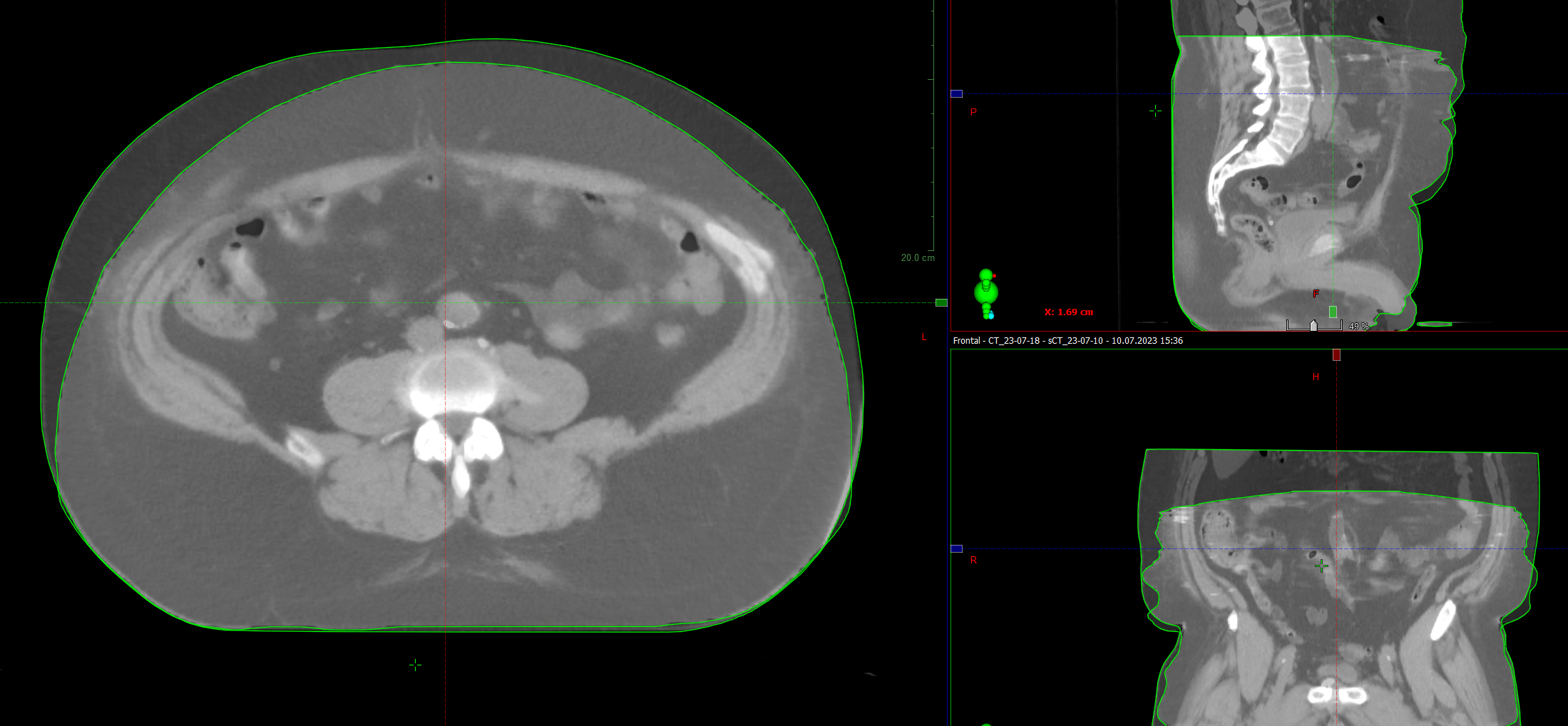


Supplementary Figure 14. Example of patient with large BMI on blended view of sCT-Dixon (left) and sCT- CT (right). The two body contours are highlighted in green to show the difference anteriorly. Patient not included in the preparatory nor in the commissioning phase due to the large BMI. Windowing of the sCT and CT -125 HU/225 HU.


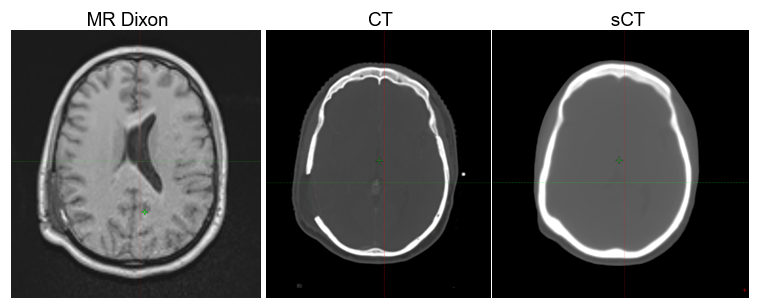

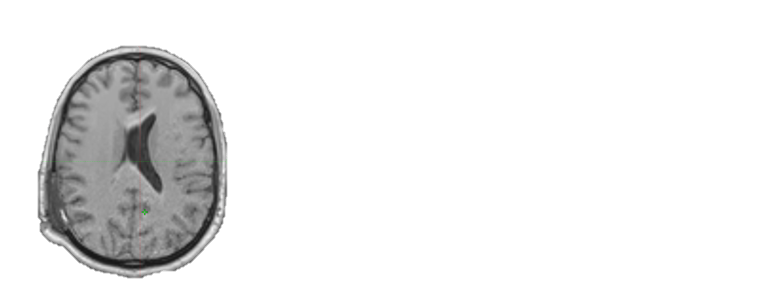

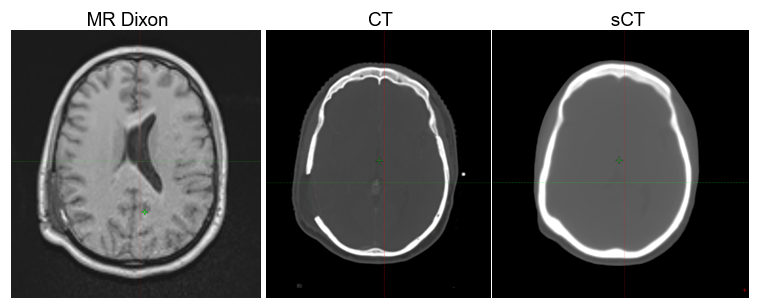


Supplementary Figure 15. Example of post-surgery open skull. Open skull is clearly visible on CT (left) and MR Dixon (centre); however, it is reconstructed as closed skull in the sCT (right). Patient not included in the preparatory nor in the commissioning phase due to the post-surgery open-skull. Windowing of the sCT and CT -400 HU/800 HU.


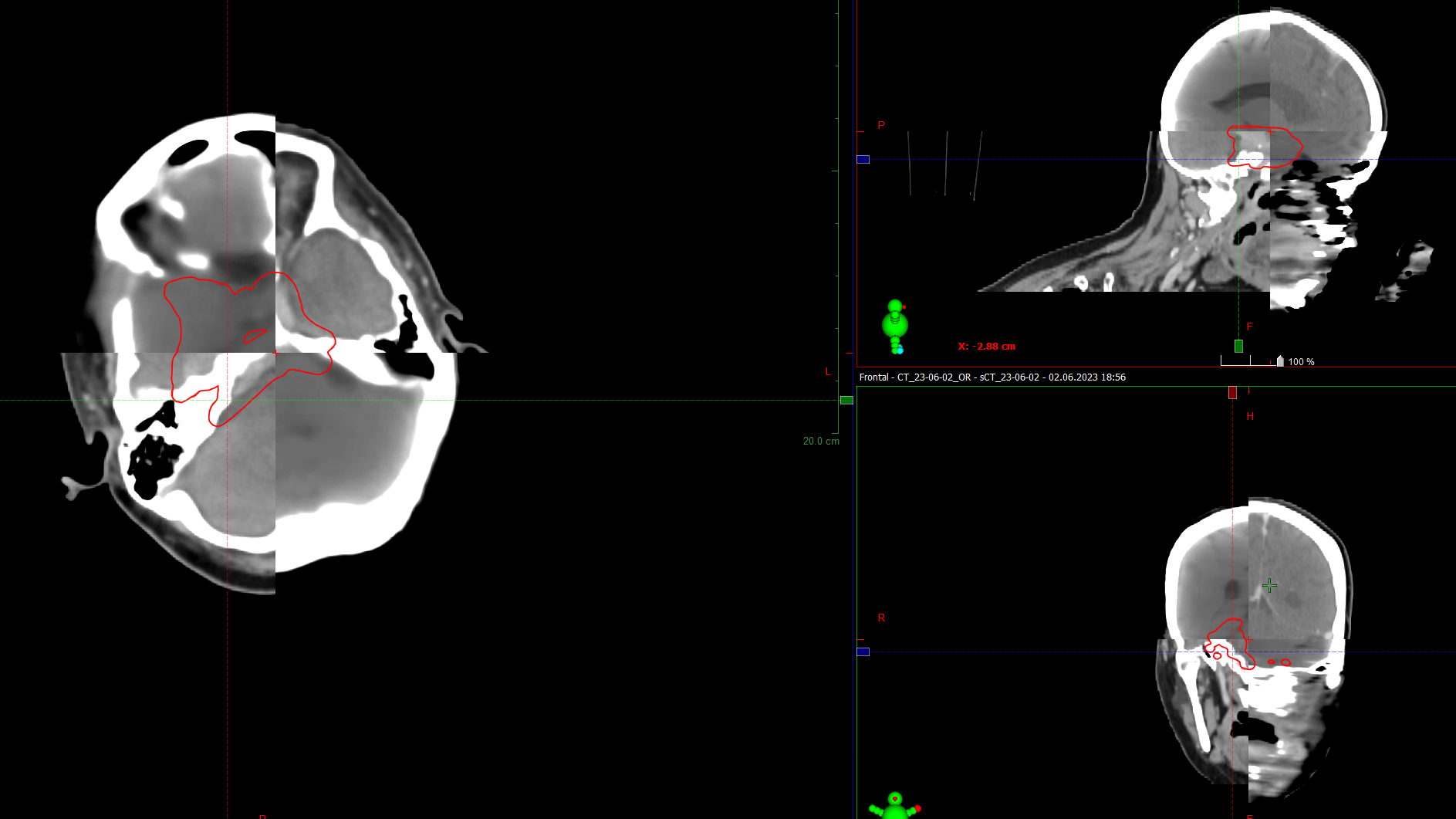

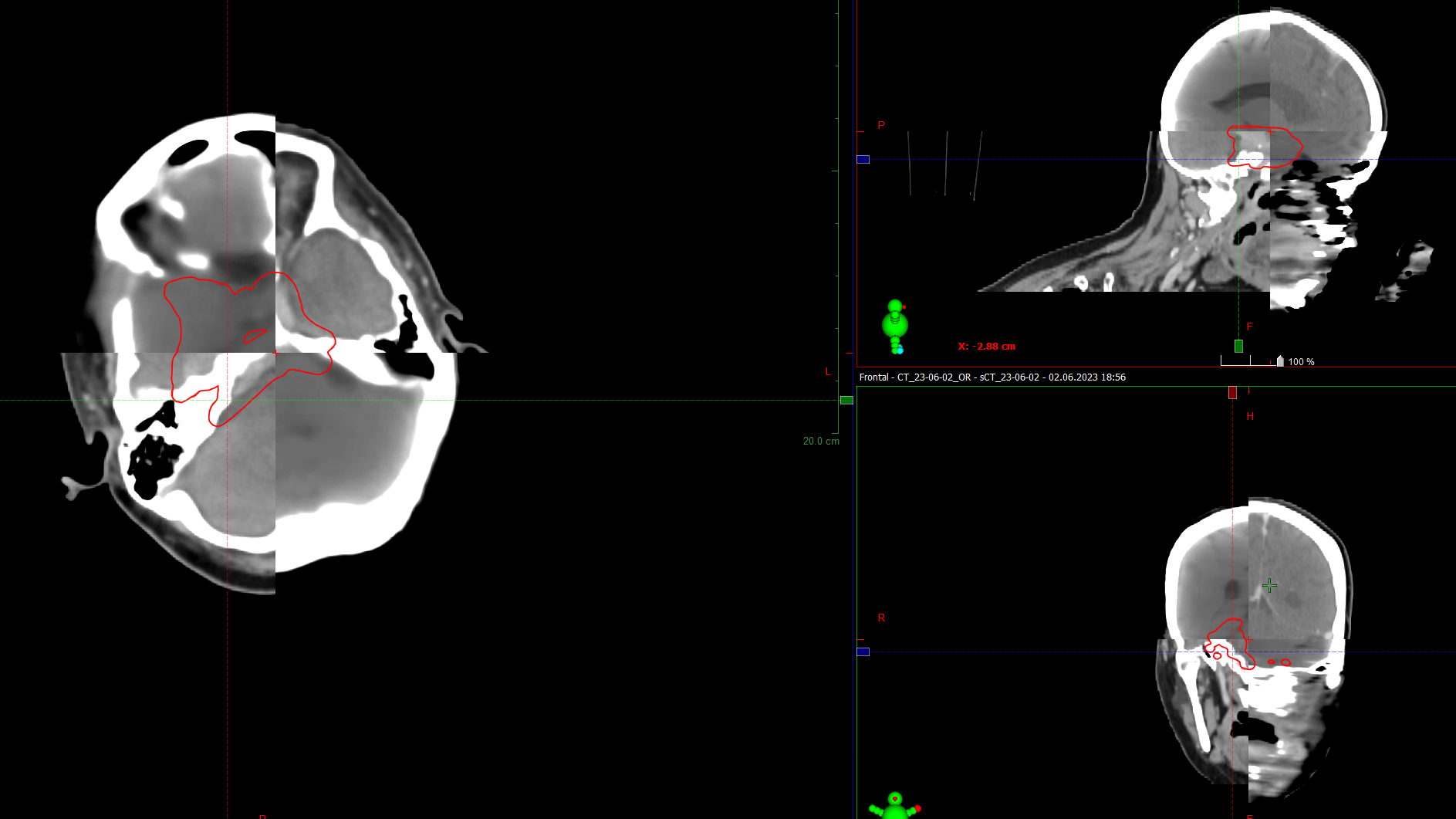

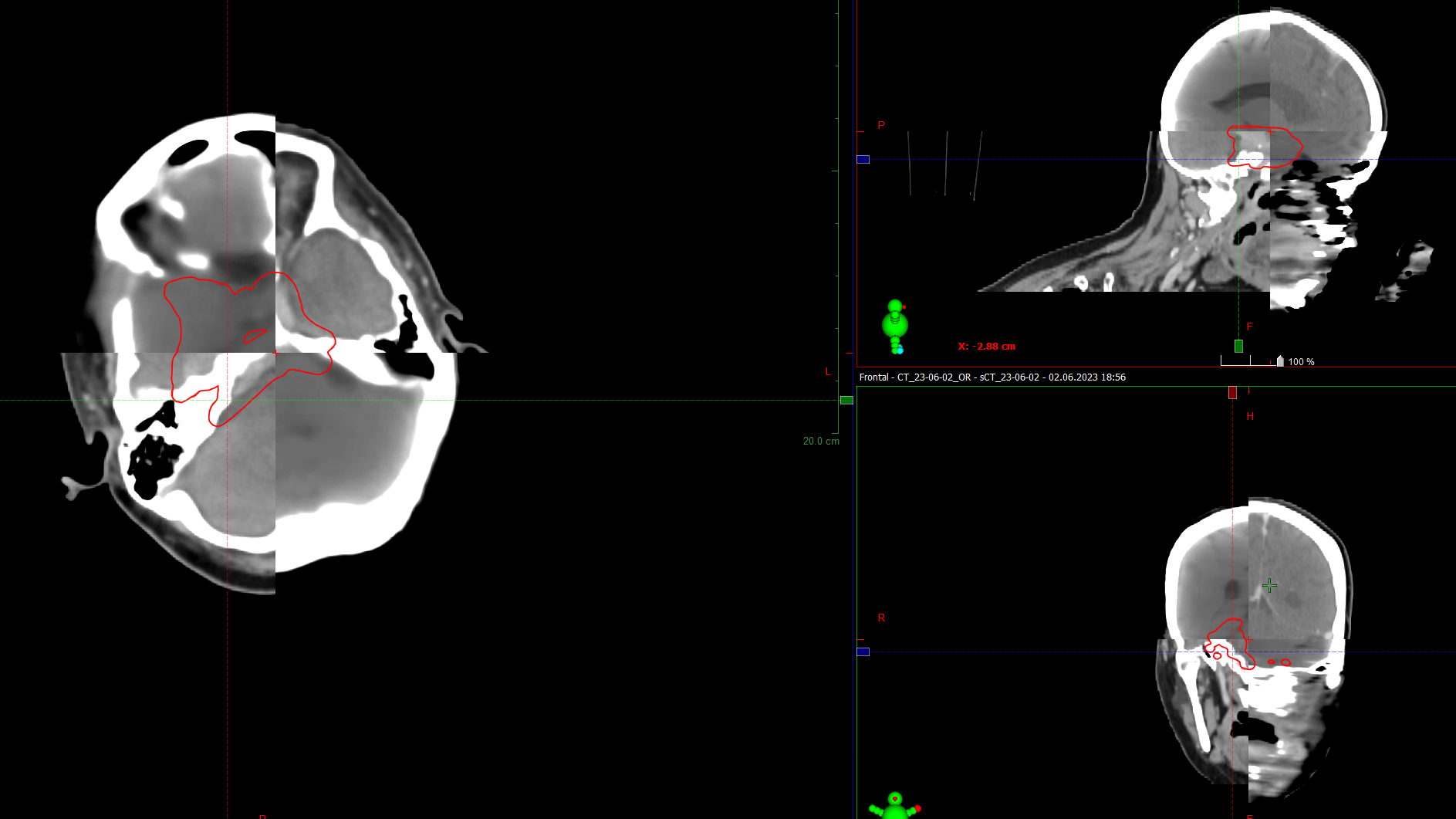


Supplementary Figure 16. Example brain patient with uncommon positioning. Patients with kyphosis struggle to lie on the couch unless a very thick headrest is placed beneath their head. Patient not included in the preparatory nor in the commissioning phase due to the positioning. Windowing of the sCT and CT -160 HU/240 HU.
